# Harnessing instability for work hardening in multi-principal element alloys

**DOI:** 10.1038/s41563-024-01871-7

**Published:** 2024-04-11

**Authors:** Bowen Xu, Huichao Duan, Xuefei Chen, Jing Wang, Yan Ma, Ping Jiang, Fuping Yuan, Yandong Wang, Yang Ren, Kui Du, Yueguang Wei, Xiaolei Wu

**Affiliations:** 1 State Key Laboratory of Nonlinear Mechanics, Institute of Mechanics, Chinese Academy of Sciences, Beijing, China; 2 School of Engineering Science, University of Chinese Academy of Sciences, Beijing, China; 3 Shenyang National Laboratory for Materials Science, Institute of Metal Research, Chinese Academy of Sciences, Shenyang, China; 4 State Key Laboratory for Advanced Metals and Materials, University of Science and Technology Beijing, Beijing, China; 5 Department of Physics, Centre for Neutron Scattering, City University of Hong Kong, Kowloon, Hong Kong, China; 6 Department of Mechanics and Engineering Science, College of Engineering, Peking University, Beijing, China

**Keywords:** Mechanical properties, Metals and alloys

## Abstract

The strength–ductility trade-off has long been a Gordian knot in conventional metallic structural materials and it is no exception in multi-principal element alloys. In particular, at ultrahigh yield strengths, plastic instability, that is, necking, happens prematurely, because of which ductility almost entirely disappears. This is due to the growing difficulty in the production and accumulation of dislocations from the very beginning of tensile deformation that renders the conventional dislocation hardening insufficient. Here we propose that premature necking can be harnessed for work hardening in a VCoNi multi-principal element alloy. Lüders banding as an initial tensile response induces the ongoing localized necking at the band front to produce both triaxial stress and strain gradient, which enables the rapid multiplication of dislocations. This leads to forest dislocation hardening, plus extra work hardening due to the interaction of dislocations with the local-chemical-order regions. The dual work hardening combines to restrain and stabilize the premature necking in reverse as well as to facilitate uniform deformation. Consequently, a superior strength-and-ductility synergy is achieved with a ductility of ~20% and yield strength of 2 GPa during room-temperature and cryogenic deformation. These findings offer an instability-control paradigm for synergistic work hardening to conquer the strength–ductility paradox at ultrahigh yield strengths.

## Abstract (web summary)

Harnessing premature necking produces a rapid multiplication of dislocations to interact with local chemical orders for work hardening in VCoNi alloy, achieving ductility of 20% and yield strength of 2 GPa during room-temperature and cryogenic deformation.

## Main

Constantly promoting yield strength is a symbol of progress in advanced high-strength metallic materials^1^. However, a gain in yield strength is normally accompanied by a sacrifice in ductility^2,3^. In general, our goal is to raise the yield strength as much as we can and retain ductility as much as possible. Ductility depends on the work-hardening ability arising from dislocations to interact among themselves for the conventional forest hardening and with diverse microstructural heterogeneities to trigger further work hardening^4^, including nanoprecipitates^5^, nanotwins^6^ and heterointerfaces^7^, along with martensitic transformation^8^. The prerequisite for work hardening is dislocation production and accumulation. However, the higher the yield strength, the more difficult it will be to produce and even store dislocations in microstructures. This becomes pronounced particularly in alloys with an ultrahigh yield strength (UHYS), usually around 2 GPa.

Recently, respectable ductility is achieved in some conventional alloys and emerging multi-principal element alloys (MPEAs) with UHYS^8–13^. The low forest-hardening ability is typical of tensile deformation due to the far-inadequate dislocation production. To further intensify work hardening, additional contribution is indispensable by either martensitic transformation^8,9^ or nanoprecipitation^10–13^ or their combination^8^, promoting to replenish and accumulate the dislocations inside grains. Otherwise, irreversible necking will prematurely happen. Meanwhile, the aforementioned UHYS alloys are either metastable (apt to martensitic transformation^8^) or full of nanoprecipitates of extremely high density^10–13^. However, it is common in most alloys with neither nanoprecipitates nor martensitic transformation. Work hardening remains unsolved if these alloys are strengthened to UHYS levels.

Here we present a unique way of work hardening that harnesses premature necking during Lüders banding to induce the rapid multiplication of dislocations. The VCoNi MPEA is selected as the prototype^14–16^, having the local-chemical-order (LCO) regions as built-in heterogeneities^17–22^. We show that in addition to forest dislocation work hardening, the dislocations further interact with the LCO regions, promoting dislocation accumulation in ultrafine grains (UFGs) and consequently contributing to additional work hardening. Dual hardening effects restrain and stabilize the premature necking in reverse and then promote large ductility. Our results show that the LCO regions can serve as an alternative to nanoprecipitates, offering an opportunity to design and achieve unprecedented mechanical properties.

## Microstructure characterization

**Fig. 1:**
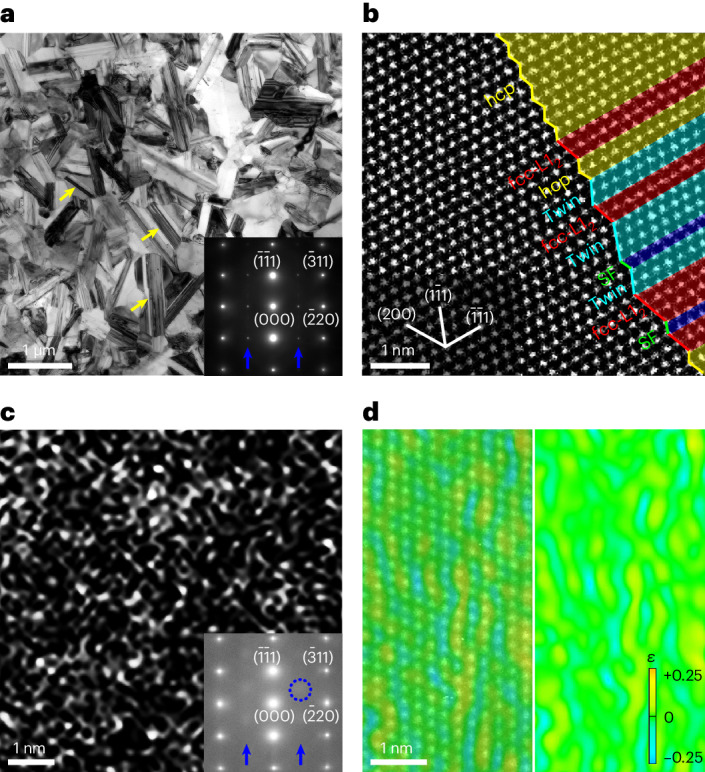
Microstructure of VCoNi MPEA. **a**, Bright-field TEM image of equiaxed fcc grains. The yellow arrows denote L1_2_ plates. The inset shows the SAED pattern with the [112] zone axis. The blue arrows indicate the extra reflections by L1_2_. **b**, HAADF lattice image of one L1_2_ plate, showing the nano-lamellae of fcc, twin and SFs, along with hcp-structured thin layers. Zone axis, [011]. **c**, Energy-filtered TEM dark-field image showing the LCO regions. The inset shows the SAED pattern. Zone axis, [112]. The blue arrows indicate the diffuse discs of superlattice scattering by LCO regions (one is circled). **d**, Overlay of two reconstructed lattice images of fcc lattice and LCO regions in green and yellow, respectively (left). GPA map of the data in the left panel (right). The alternative yellow and blue strips indicate the strain-field contrast of LCO regions. In the scale bar, *ε* denotes the lattice strain.

We obtained the microstructure of face-centred-cubic (fcc)-structured UFGs by thermomechanical processing at 1,173 K for 150 s in the VCoNi alloy, similar to that reported previously^20^. The fcc UFGs are equiaxed and have an average size ($\bar{d}$) of 0.42 μm in the transmission electron microscopy (TEM) image (Fig. 1a). The plates of ordered L1_2_ intermetallic compound (yellow arrows) appear in fcc grains. Figure 1a (inset) shows the superlattice spots (blue arrows) induced by L1_2_ in the selected-area electron diffraction (SAED) pattern. The L1_2_ plate consists of nano-lamellae, including the fcc structure, twin and stacking fault (SF), along with a minor amount of hexagonal close-packed (hcp) structure as seen in the high-angle annular dark-field (HAADF) image (Fig. 1b) and corresponding energy-dispersive X-ray spectroscopy mapping (Supplementary Fig. 1 and Supplementary Notes 1 and 2).

The fcc grains also contain the LCO regions^18,20^ (Fig. 1c). Recently, the LCO regions have been gradually recognized as inherent heterogeneities in some MPEAs^17–22^. It is noted that we acknowledge the uncertainties and ongoing exploration on the structure of LCO regions^23^, which, however, should not influence the main conclusions here based on the evidence obtained by current techniques. The LCO regions are visible in fcc grains by using energy-filtered dark-field imaging (Fig. 1c). The statistical average size and volume fraction is 0.65 nm and 4%, similar to those previously reported^20^. By using the atomic-scale geometric phase analysis (GPA) mapping^24^, strain fields are determined by reconstructing two lattice images of the fcc matrix and LCO regions via individual reflections in the SAED pattern (Fig. 1c, inset) and overlaying them (Fig. 1d, left). The LCO region shows the strain-field contrast as an alternate yellow and blue stripe on the green fcc matrix in the GPA map^20^ (Fig. 1d, right).

## Mechanical properties

**Fig. 2:**
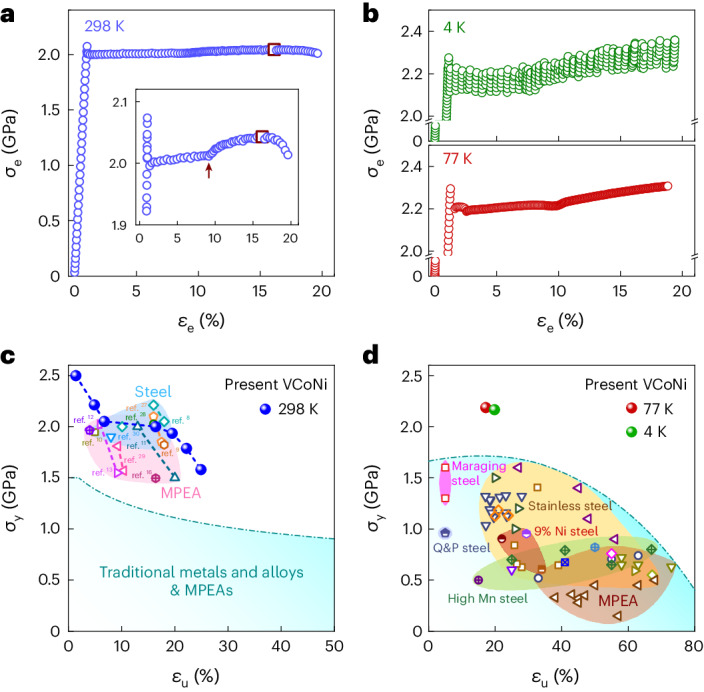
Mechanical properties and strength–ductility balance at room and cryogenic temperatures. **a**,**b**, Tensile engineering stress–strain (*σ*_e_–*ε*_e_) curve at room (298 K) (**a**) and cryogenic (4 and 77 K) (**b**) temperatures. The square symbol indicates the ductility at the ultimate tensile strength. The inset in **a** shows an enlarged curve indicating the LB propagation followed by uniform elongation under work hardening. The arrow indicates the LB end. **c**,**d**, Yield strength (*σ*_y_) and ductility (*ε*_u_) balance at 298 K (**c**) and 77 and 4 K (**d**). Other data with UHYS are shown for comparison in **c**, including MPEAs (pink areas) and steels (blue area).

The UFG VCoNi shows a respectable ductility (*ε*_u_) of 16% and yield strength (*σ*_y_, lower yield point) of 2 GPa at room temperature (298 K), as evident in the tensile engineering stress–strain (*σ*_e_–*ε*_e_) curve (Fig. 2a). Both *ε*_u_ and *σ*_y_ increase to around 20% and 2.2 GPa, respectively, during cryogenic deformation at liquid-helium (4 K) and liquid-nitrogen (77 K) temperatures (Fig. 2b). Here *ε*_u_ is conventionally regarded as the sum of the Lüders band (LB) propagation strain and the uniform strain when the LB arises^8^. The simultaneous increase in both *ε*_u_ and *σ*_y_ is the common feature during cryogenic deformation in fcc metals and alloys^25,26^. Importantly, the yield drop followed by a propagating LB consistently appear as an initial plastic response in three tensile curves (Fig. 2a,b (insets) and Supplementary Note 3).

**Extended Data Fig. 1:**
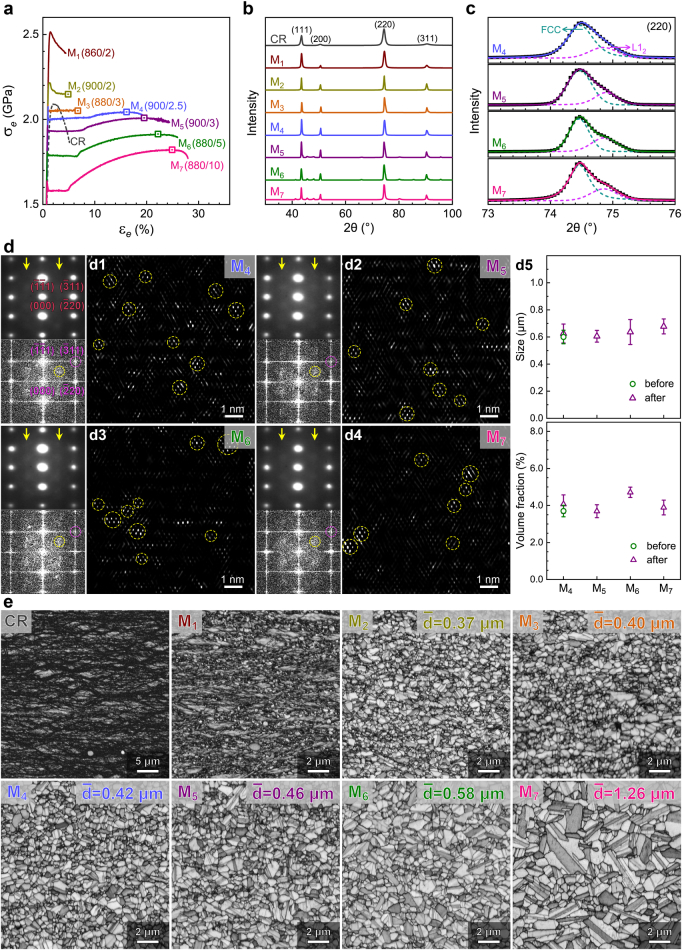
Tensile properties and microstructures at 298 K corresponding to seven blue data points of various yield strengths as shown in Fig. 2c, showing the consistent phase constituents in all microstructures. (**a**) Tensile engineering stress-strain curves. The tensile curve after cold rolling (CR) is also shown for comparison. Seven curves, corresponding to their microstructures, are labeled by M_*x*_, where subscript *x* from 1 to 7 indicates various temperature and period for recrystallization annealing as shown in bracket (number °C / time minutes). The short period annealing enables to maintain the consistent phase constituents in each sample. Here, curve M_4_ is that shown in Fig. 2a. Curves from M_2_ to M_7_ show the common tensile response, that is, the LB propagation followed by uniform deformation. Curves M_2_ and M_3_ show the direct fracture of samples during the LB propagation, without uniform deformation. Curve M_1_, along with curve CR, shows that necking happens soon after yielding, with hardly any ductility. (**b, c**) X-ray diffraction spectra in various microstructures, together with the separation of asymmetric peak that consists of two overlapping peaks of fcc and ordered L1_2_ lattice. The volume fraction of L1_2_ was determined in a range of 13%-15% in all microstructures. (**d**) LCO regions after tensile deformation in various microstructures observed by using the method proposed previously^20^. **d1** (M_4_): nano-beam diffraction pattern (upper left) and Fast Fourier Transform (FFT) pattern (lower left), showing the signature diffuse disks induced by LCO regions, labeled by yellow arrows and dash circles. The inverse FFT lattice image (right panel) was reconstructed by diffuse disks, showing LCO regions (yellow circles). **d2-d4**: corresponding inverse FFT images in M_5_-M_7_, showing the LCO regions (yellow dash circles). **d5**: average size and volume fraction of LCO regions (upper and lower panel, respectively) after tensile deformation. LCO regions in M_4_ are unchangeable in mean sizes and densities before and after tensile deformation. Sizes and volume fraction keep consistent in four microstructures after tensile deformation. The mean values ± standard error of the mean are presented based on 20 lattice images of each sample used to derive statistics. (**e**) SEM EBSD images in various microstructures. The statistical average grain size is shown in each image.

Tailoring the microstructure gains a series of (*σ*_y_, *ε*_u_) balances at 298 K (Fig. 2c, seven blue balls). Extended Data Fig. 1 shows the corresponding *σ*_e_–*ε*_e_ curves, along with the detailed microstructural characterization. These (*σ*_y_, *ε*_u_) balances are on par with those in other MPEAs and steels with UHYSs^8–13,27–30^. The cryogenic (*σ*_y_, *ε*_u_) synergy is prominent (Fig. 2d). It indicates that the VCoNi MPEA has suppressed the ductile-to-brittle transition that typically jeopardizes conventional steels usually with a body-centred-cubic and/or martensite phase. In this sense, the VCoNi MPEA combines the signature attribute of established alloys: desirable ductility in fcc alloys, which can be sustained to cryogenic temperatures, along with UHYS values known for advanced high-strength steels. Therefore, the VCoNi MPEA is a good candidate for cryogenic applications that need high strength and large ductility.

## Premature necking and plastic responses

**Fig. 3:**
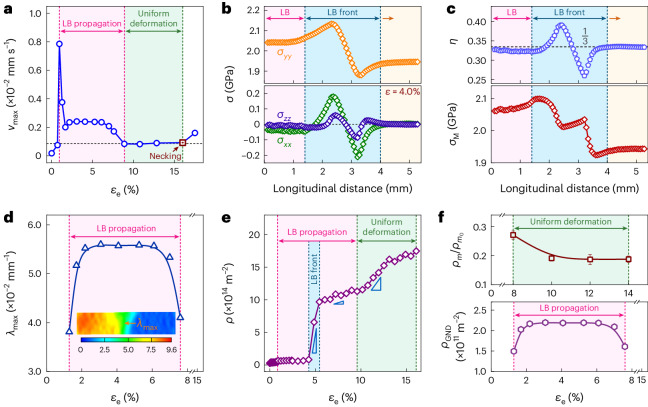
Premature necking at the LB front, plastic responses and dislocation multiplication. **a**, Maximum lateral shrinkage rate (*v*_max_) induced by premature necking at the LB front, showing the steep rise to drop followed by the plateau during LB propagation. The dashed line indicates the shrinkage rate during uniform deformation. The square symbol indicates the onset of final diffuse necking. **b**, Change in stress state in the LB front region, showing three stress components at a tensile strain of 4%. The top and bottom panels show *σ*_*yy*_, *σ*_*xx*_ and *σ*_*zz*_ along the length, width and thickness directions of the tensile sample, respectively. The arrow indicates the LB propagation direction towards the deformation-free gauge section. **c**, Change in stress triaxiality (*η*; top) and von Mises stress (*σ*_M_; bottom) in the LB front region. The dashed line (top) shows the standard *η* value (1/3) during uniform deformation. **d**, Maximum strain gradient (*λ*_max_) at the LB front measured by the DIC test. The inset shows a snapshot of the full-field strain distribution at a strain of 4.0%. The arrow shows *λ*_max_ at the LB front. **e**, Evolution of dislocation density *ρ* as a function of tensile strain measured by means of the synchrotron-based high-energy XRD in situ tensile testing. Note the rapid multiplication of *ρ* from the initial 2.6 × 10^13^ to 9.7 × 10^14^ m^−2^ at the LB front. **f**, Evolution of mobile dislocation density during uniform deformation (top) and GND density during LB propagation (bottom). For the data in the top panel, mean values ± standard error of the mean are presented on the basis of three stress-relaxation tensile tests.

We first probed into the deformation physics behind Lüders banding in the present UHYS UFG. The key finding is the localized lateral shrinkage that appears at the LB front obtained from the digital-image-correlation (DIC) tensile test (Supplementary Fig. 2a1,2). The localized shrinkage is characterized by the shrinkage rate, *v* = (*w*_t_ – *w*_i_)/Δ*t*, where *w*_i_ and *w*_t_ are the initial and transient widths of the gauge section, respectively, and Δ*t* is the time interval. The maximum value, *v*_max_, locates at the LB front (Fig. 3a). Here *v*_max_ rapidly rises to the peak, then drops soon after yield drop and finally reaches a plateau during LB propagation. The following plateau (Fig. 3a, horizontal dashed line) is due to the Poisson’s ratio effect during uniform elongation. Both *v*_max_ peak and plateau during LB propagation are eight and two times that during uniform deformation, respectively, and even higher than that when final necking begins. This indicates that the localized shrinkage is essentially necking that keeps happening during LB propagation. Namely, premature necking has already happened at the LB front, yet with no sign left in the engineering stress–strain curve, unlike usual necking that shows a decrease in flow stress. Further, *v*_max_ shows a rapid drop from the peak followed by a stable plateau. This suggests that work hardening has been induced during LB propagation, restraining the premature necking (Supplementary Note 4).

We then focus on two plastic responses inevitably induced by premature necking. The first is triaxial stress due to geometrical inhomogeneities at the LB front^31^. Three stress components are determined (Fig. 3b), that is, *σ*_*xx*_, *σ*_*zz*_ and *σ*_*yy*_, using finite-element-method (FEM) simulations (Supplementary Fig. 3a). This indicates a change in the stress state from uniaxial to triaxial in the LB front region, in contrast to an approximately uniaxial state on both sides. Further, the triaxial stress leads to an elevated von Mises stress (*σ*_M_) in the LB front region (Fig. 3c, bottom). The triaxial stress state is characterized by the triaxiality parameter (*η*) defined as the hydrostatic press divided by *σ*_M_ (ref. ^32^). Here *η* varies from the least value of 0.26 to the largest value of 0.39 (Fig. 3c, top), relative to *η* of $\tfrac{1}{3}$ by uniform shrinkage. Both maximum and minimum *η* are constant during LB propagation (Supplementary Fig. 3), indicating a stable triaxial stress state. The second is the strain gradient (*λ*) (ref. ^33^). Here *λ* arises from the macroscopic plastic incompatibility between the LB front region and remaining elastic section. Also, *λ* is defined as $\tfrac{\partial \varepsilon }{\partial x}$, where *ε* is the longitudinal strain and *x* is the distance from the LB front. The maximum value, *λ*_max_, is located at the LB front and remains unchangeable during LB propagation (Fig. 3d). Both triaxial stress and strain gradient will facilitate to trigger dislocation production at the LB front.

## Dislocation multiplication

**Extended Data Fig. 2:**
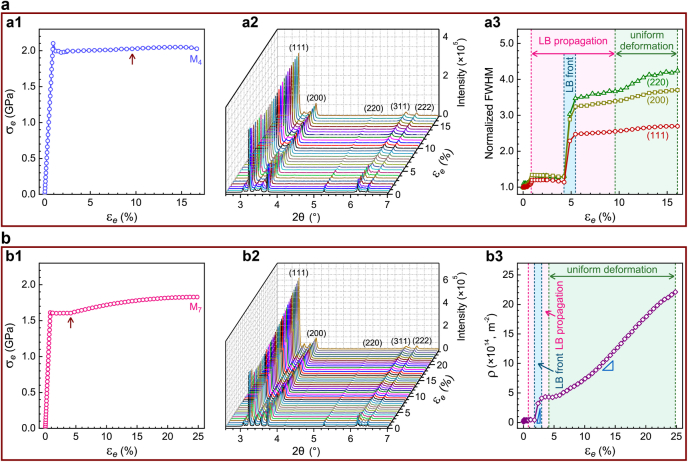
Dislocation multiplication in two microstructures during tensile deformation measured by the synchrotron-based high-energy X-ray diffraction *in situ* tensile testing. (**a**) Results in M_4_ as shown in Fig. 1a with yield strength of 2 GPa as shown in Fig. 2a. **a1**: The *σ*_*e*_−*ε*_*e*_ curve by in-situ testing. Arrow: the end of LB propagation. **a2**: Change of diffraction intensities with tensile strains. **a3**: Evolution of normalized full width at half maximum (FWHM) for (111), (200), and (220) peaks in fcc lattices as a function of tensile strain. (**b**) Corresponding results in M_7_ with yield strength of 1.6 GPa as shown in Extended Data Fig. 1a. **b1**: The *σ*_*e*_−*ε*_*e*_ curve by in-situ testing. Arrow: the end of LB propagation. **b2**: Change of diffraction intensities with tensile strains. **b3**: Change of dislocation density (*ρ*) during tensile deformation. Note that *ρ* increases from the initial 1.9 × 10^13^ m^−2^ to 4.1 × 10^14^ m^−2^ at first when the LB arrives, then to 4.3 × 10^14^ m^−2^ by the end of LB propagation, and finally to 2.2 × 10^15^ m^−2^ after uniform deformation (Fig. 3e). The share of the increment in *ρ* during the LB propagation is 17% of total increment. Note the mass formation of dislocations during uniform deformation, in sharp contrast to what happened in M_4_ (Fig. 3e).

We investigated the dislocation production at first by means of the synchrotron-based high-energy X-ray diffraction (XRD) in situ tensile testing (Extended Data Fig. 2a). The dislocation density (*ρ*) increases from the initial 2.6 × 10^13^ to 9.7 × 10^14^ m^−2^ once the LB front arrives and then to 1.2 × 10^15^ m^−2^ by the end of LB propagation; finally, it reaches 1.7 × 10^15^ m^−2^ when uniform deformation ends (Fig. 3e). The share of increment of *ρ* (Δ*ρ*) at the LB front is noteworthy, reaching ~80% during the LB propagation. Meanwhile, the dislocation multiplication rate ($\dot{\rho }$), calculated as Δ*ρ*/Δ*t*, is 4.6 × 10^13^ m^−2^ s^−1^ at the LB front, which is 12 and 6 times quicker than that during the later LB propagation and uniform deformation, respectively. It follows that the dislocations rapidly multiply at the LB front where premature necking happens. Namely, necking promotes dislocation multiplication. Triaxial stress, along with an enhanced von Mises stress, facilitates to activate more slip systems and sources inside grains such that the dislocations multiply at a more rapid speed^34^. These dislocations are forest dislocations, also called statistically stored dislocations (SSDs)^35^. Most of them are initially mobile. We then measured the change in mobile dislocations density (${\rho }_{{\rm{m}}}/{\rho }_{{{\rm{m}}}_{0}}$) by stress-relaxation testing (Supplementary Fig. 4)^36^, where ${\rho }_{\mathrm{m}_{0}}$ and *ρ*_m_ are the initial and transient mobile dislocation density, respectively. The mobile dislocations, as the precondition of LB initiation^8,31^, accommodate not only highly accumulated strains at the LB front (Supplementary Fig. 2a4, top) but also uniform strains. Also, ${\rho }_{\mathrm{m}}/{\rho }_{\mathrm{m}_{0}}$ drops at first and then levels off during uniform deformation (Fig. 3f, top). This indicates the formation of mobile dislocations during LB propagation. We finally estimate the density of geometrically necessary dislocations (GNDs), *ρ*_GND_, as *λ*/*b* (refs. ^37,38^), where *b* is the magnitude of Burgers vector (0.255 nm). The GNDs compensate for strain incompatibilities^33^, particularly offering local stress and extra work hardening^7,39^. Also, *ρ*_GND_ is 2.5 × 10^11^ m^−2^ during LB propagation (Fig. 3f, bottom), consistent with previously reported values^40^.

**Fig. 4:**
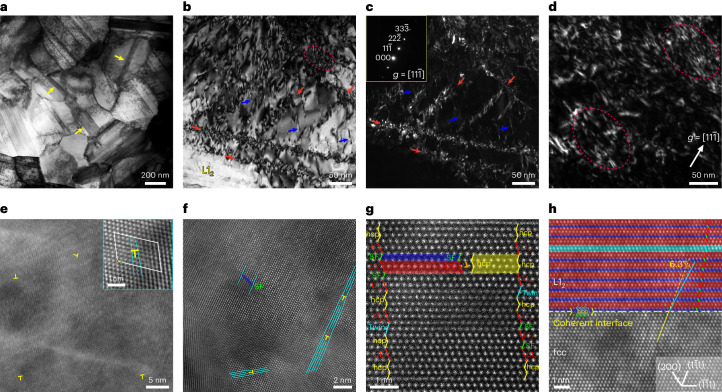
Dislocation behaviours in fcc grains and L1_2_. **a**, Dislocation production inside fcc grains at the LB front after interrupted tensile testing at a strain of 5% (298 K). **b**,**c**, Weak-beam TEM bright-field (**b**) and dark-field (**c**) images of dislocations (298 K). The red arrows show the sub-boundaries of the dislocation cell. The blue arrows show the SFs. The dashed circle indicates the dislocation tangle. The bright contrast in **c** indicates dislocations. The inset in **c** shows the weak-beam **g**/3**g** pattern, with **g** = [11$\bar{1}$]. **d**, Weak-beam dark-field image. The dashed circle indicates dislocation tangles. **e**,**f**, High-resolution lattice images in fcc grains after tensile deformation at 298 and 77 K, respectively, showing dislocations. Zone axis, [110]. The ‘T’ symbols indicate extra half-plane of edge dislocation. The inset in **e** shows the Burgers circuit encircling one dislocation (T) having a Burgers vector of ½[1$\bar{1}$0]. **g**, HAADF image, showing nano-lamellar L1_2_. Zone axis, [011]. The ⊥ symbol indicates the core of the Shockley partial. The red, blue, green and yellow lines indicate fcc-L1_2_, twin, SF and hcp lattice, respectively. **h**, Lattice image showing the coherent L1_2_/fcc-phase interface. Note the lattice rotation by 6.3° in L1_2_.

**Extended Data Fig. 3:**
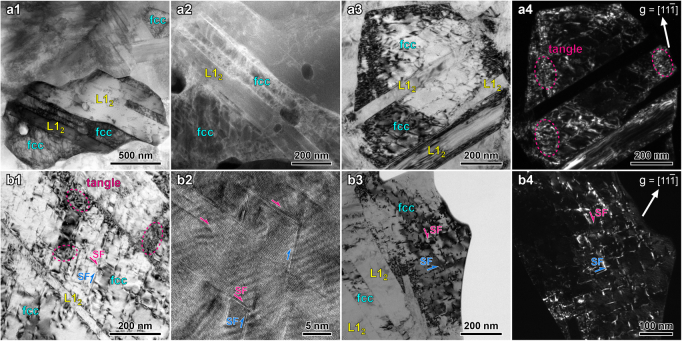
Dislocation behaviors after cryogenic deformation at 77 K and 4 K by TEM observations. (**a**) Dislocations at 77 K. **a1,a2**: Bright-field diffraction contrast image and enlarged STEM HAADF dark-field image, showing the high-density dislocations in fcc grains. A few particles are σ compounds, which contribute little to work hardening due to very small fraction and large sizes. **a3,a4**: Bright-field and weak beam dark-field TEM image, showing the dislocation tangles in fcc grains. Dash circles in **a4**: dislocation tangles. (**b**) Dislocations at 4 K. **b1, b2**: Bright-field TEM image and corresponding HAADF lattice image. Arrows: SFs. Dash circles: dislocation tangles. **b3,b4**: Bright-field and corresponding weak beam dark-field TEM image, showing SFs of high density (arrows), along with entangled dislocations.

The dislocation behaviours were further investigated by site-specific TEM observations after an interrupted tensile testing. The dislocations are rapidly produced and accumulated in most grains once the LB front arrives (Fig. 4a and Supplementary Fig. 5), forming dislocation tangles and low-angle sub-boundaries (Fig. 4b,c, red arrows), along with small amounts of SFs (blue arrows). The weak-beam dark-field TEM image shows that dislocations prefer to tangle up and reside at the dislocation sub-boundaries (Fig. 4c), leaving less and less room in the grains to further accumulate dislocations. The dominant pattern is entangled dislocations by the end of LB propagation (Fig. 4d), which interact with and capture the remaining mobile dislocations to make them immobile. Besides, the high-resolution TEM lattice image shows a local dislocation density of 2.8 × 10^15^ m^−2^ after tensile deformation at 298 K (Fig. 4e), with the same order as that by XRD measurements (Fig. 3e). Also, *ρ* increases after cryogenic deformation at 77 K (Extended Data Fig. 3a1–4), with *ρ* as high as 8.8 × 10^15^ m^−2^ (Fig. 4f). The increased *ρ* value facilitates the simultaneous increase in strength and ductility (Fig. 2b). The large amount of SFs are visible after cryogenic deformation at 4 K (Extended Data Fig. 3b1–4). This is probably the reason for the serration flow at extremely low temperatures^41^.

We also investigated the dislocation behaviours in the L1_2_ plate. The electron concentration (*e*/*a*) is 8.43, which is calculated according to the chemical compositions of fcc-L1_2_ (Supplementary Fig. 1a). Therefore, the L1_2_ plate is hardly deformable based on the *e*/*a* criterion^14^. As expected, only a few Shockley partials are formed during plastic deformation and one is labelled by Τ (Fig. 4g), with the Burgers vector of the $\tfrac{1}{6} < 2\bar{1}\bar{1} >$ type. It is observed further that the (1$\bar{1}$1) planes in fcc-L1_2_ rotate by an angle of 6.3° (*φ*) to induce a shear strain (~*tgφ*) of 11% (Fig. 4h), that is, an equivalent strain of ~6%, whereas the L1_2_/fcc-phase interface remains coherent. Accordingly, the L1_2_ plates mainly accommodate the microscopic strains by lattice rotation and contribute little to work hardening (Supplementary Note 5).

## Work hardening

**Fig. 5:**
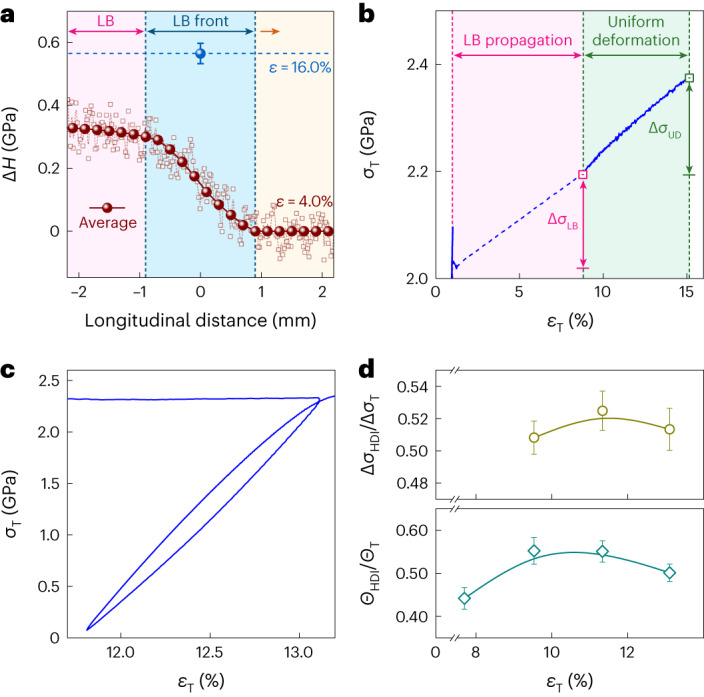
Work hardening. **a**, Gradient microhardness increment (Δ*H*) in the LB front area measured in the sample after an interrupted tensile strain of 4%. The arrow shows the direction of LB propagation. The dashed line shows the mean Δ*H* after tensile deformation. The mean values and error bands of the mean are presented on the basis of microhardness tests conducted at five positions with equal spacing along the width of the gauge section. **b**, Tensile true stress–strain (*σ*_T_−*ε*_T_) curve corresponding to the engineering (*σ*_e_−*ε*_e_) curve shown in Fig. 2a. The dashed line merely connects the start and end of LB propagation. Here Δ*σ*_LB_ and Δ*σ*_UD_ denote the increments in flow stress during LB propagation and uniform deformation, respectively. **c**, Mechanical hysteresis loop during an unload–reload cycle at an unload strain of 13.1%. **d**, Ratio of Δ*σ*_HDI_ to Δ*σ*_T_ (top) and that of *Θ*_HDI_ to *Θ*_T_ (bottom). Here *σ*_HDI_ and *Θ*_HDI_ denote the HDI stress and HDI-stress-induced hardening rate, respectively, and *σ*_T_ and *Θ*_T_ denote the true stress and global work-hardening rate, respectively. The mean values ± standard error of the mean are presented on the basis of three unload–reload tensile tests.

We now place emphasis on work hardening that plays a decisive role in the restraint of premature necking. First, a microhardness increment (Δ*H*) appears as a gradient in the LB front region (Fig. 5a), well consistent with the rapid increase in *ρ* at the LB front (Fig. 3e and Supplementary Fig. 5). This signals that work hardening begins once the LB front arrives. Second, the cumulative work hardening can be described by the increment in flow stress as strain increases in the true stress–strain (*σ*_T_−*ε*_T_) curve (Fig. 5b). The increment during LB propagation, namely, Δ*σ*_LB_, and that during uniform deformation, that is, Δ*σ*_UD_, are 182 and 173 MPa, respectively. During these two stages, the corresponding forest stress increments induced by SSDs (Fig. 3e), Δ*σ*_SSDs_, are only 110 and 80 MPa, which are calculated by the Taylor hardening law, that is, ${\Delta \sigma }_{{{\rm{SSDs}}}}=M\alpha \mu b(\sqrt{{\rho }_{{\rm{e}}}}-\sqrt{{\rho }_{{\rm{s}}}})$, where *M* is the Taylor factor (3); *α* is a constant (0.2); *μ* is the shear modulus (72 GPa); and *ρ*_s_ and *ρ*_e_ are the dislocation densities at the start and end of each stage, respectively. A non-negligible gap exists between the flow stress increment and forest stress increment in the two stages, which are 72 and 93 MPa, accounting for 40% and 54% in the flow stress increment, respectively. This indicates that the forest stress is only a part of the flow stress. Namely, forest dislocation hardening alone is inadequate to support the global work hardening in two stages.

We then find out the additional stress to compensate for the aforesaid stress gap by conducting an interrupted tensile test (Supplementary Fig. 6). An important finding is the mechanical hysteresis loop that repeatedly occurs during each unload–reload cycle. One loop is shown at an unload strain of 13.1% (Fig. 5c). The hysteresis loop is the characteristic mechanical response, indicating that both SSDs and GNDs simultaneously contribute to plastic deformation. To be specific, GNDs will induce a heterodeformation-induced (HDI) stress (*σ*_HDI_) (refs. ^7,39^), serving as an essential supplement to Δ*σ*_SSDs_. Accordingly, the flow stress increment during uniform deformation, Δ*σ*_T_, is equal to Δ*σ*_SSDs_ plus Δ*σ*_HDI_. The measured Δ*σ*_HDI_ accounts for ~51% in Δ*σ*_T_ (Fig. 5d, top), effectively filling in the aforementioned stress gap of 54%. Further, *σ*_HDI_ produces the HDI hardening, characterized by the hardening rate *Θ*_HDI_ (∂*σ*_HDI_/∂*ε*) (refs. ^7,39^). The global hardening rate *Θ*_T_ is the sum of the hardening rates by SSDs and GNDs. It is visible that the percentage of *Θ*_HDI_ reaches 50% (Fig. 5d, bottom). These results indicate the indispensable contribution of both *σ*_HDI_ and *Θ*_HDI_ to the global flow stress and work hardening during uniform deformation. Because of the heterogeneous LB propagation, *σ*_HDI_ cannot be calculated. However, the decisive role of both *σ*_HDI_ and *Θ*_HDI_ is certain during the LB deformation. This is due to the fact that the percentage of Δ*σ*_SSDs_ (110 MPa) is only ~60% of Δ*σ*_T_ (182 MPa). Accordingly, the forest hardening combines HDI hardening to slow down and stabilize the premature necking for the LB propagation and uniform elongation.

**Fig. 6:**
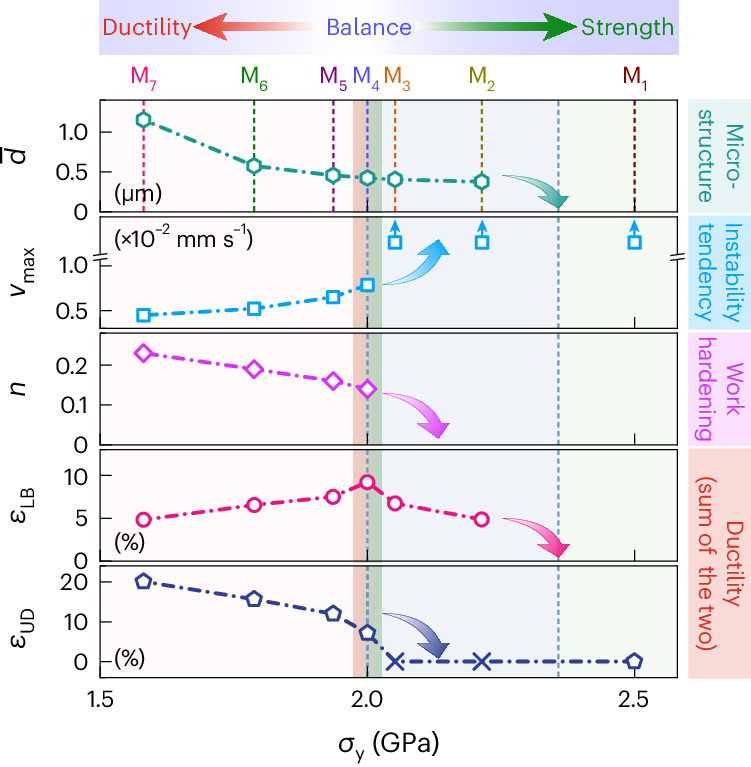
Work hardening for strength–ductility balance by harnessing premature necking. The *x* axis shows the yield strength (*σ*_y_). M_*x*_ (top) indicate the seven microstructures (*x* = 1–7, corresponding to the seven data points shown in Fig. 2c and tensile stress–strain curves (Extended Data Fig. 1a)). The *y* axis shows the mechanical properties and parameters. The symbols indicate the measured data. The arrow shows the trend. Here $\bar{d}$ indicates the average size of the fcc grains; *v*_max_, the maximum lateral shrinkage rate at the LB front; *n*, work-hardening exponent; *ε*_LB_, Lüders strain; *ε*_UD_, uniform strain. Note the lost *ε*_UD_ (labelled by ×) due to direct fracture of the sample during LB propagation in both M_2_ and M_3_.

We finally return to seven data points (Fig. 2c) to demonstrate the effect of premature necking on work hardening (Fig. 6). The corresponding microstructures have similar tensile features and phase constituents (M_1_–M_7_; Extended Data Fig. 1) but different $\bar{d}$ values in fcc grains. The mechanical parameters, including *v*_max_, *η* and *λ*, were measured and tested (Supplementary Figs. 2b and 3b). The work-hardening exponent *n* is fitted by *σ* = *k* × *ε*^*n*^, where *k* is a constant. Among them, *v*_max_ and $\bar{d}$ deserve special mention. First, *v*_max_ shows dual effects. One is to signal the onset and degree of premature necking. The other is to trigger large Δ*ρ* and $\dot{\rho }$, that is, the rapid multiplication of SSDs and GNDs. Namely, *v*_max_ arises from the lack of work hardening but produces—in reverse—the combined work hardening. In particular, the HDI hardening effectively makes up the deficiency of forest hardening (Fig. 5d, bottom). Second, $\bar{d}$ is decisive to tailor work hardening. Here $\bar{d}$ causes conflict between *v*_max_ and *n*. Specifically, the lower the value of $\bar{d}$, the larger is the *v*_max_ value and lower is the value of *n*. Also, $\bar{d}$ in M_4_ (Fig. 1a) is optimal for obtaining just the right work hardening to restrain and stabilize the LB, achieving respectable ductility along with the highest *σ*_y_ value (Fig. 2a). Yet, the decrease in $\bar{d}$ in M_2_ and M_3_ makes an increased *v*_max_ and decreased *n*. Work hardening is insufficient and fracture happens during the LB propagation. Also, *ε*_LB_ drops and *ε*_UD_ is even lost. On the contrary, an increased $\bar{d}$ in M_5_–M_7_ leads to decreased *v*_max_ and increased *n*. Compared with M_4_, M_7_ increases $\bar{d}$ from 0.42 to 1.26 μm (Supplementary Note 6). The *v*_max_ peak in M_7_ is only half that in M_4_, still maintaining an almost unchangeable *v*_max_ plateau (Supplementary Fig. 2b5). This indicates that premature necking happens in M_7_. Also, Δ*ρ* decreases to 3.9 × 10^14^ m^−2^ during the LB propagation and increases to 21.7 × 10^14^ m^−2^ during the entire tensile deformation (Extended Data Fig. 2b3), along with a decreased ${\dot{\rm{\rho}}}$ value to 1.9 × 10^13^ m^−2^ s^−1^ at the LB front. All these changes are ascribed to an increased $\bar{d}$ value in M_7_, leading to a decreased premature necking tendency. As a result, *ε*_LB_ drops and *ε*_UD_ rises, thereby increasing ductility. It follows that harnessing the premature instability is practical to achieve superior yield strength–ductility synergy.

## Discussion and concluding remarks

**Extended Data Fig. 4:**
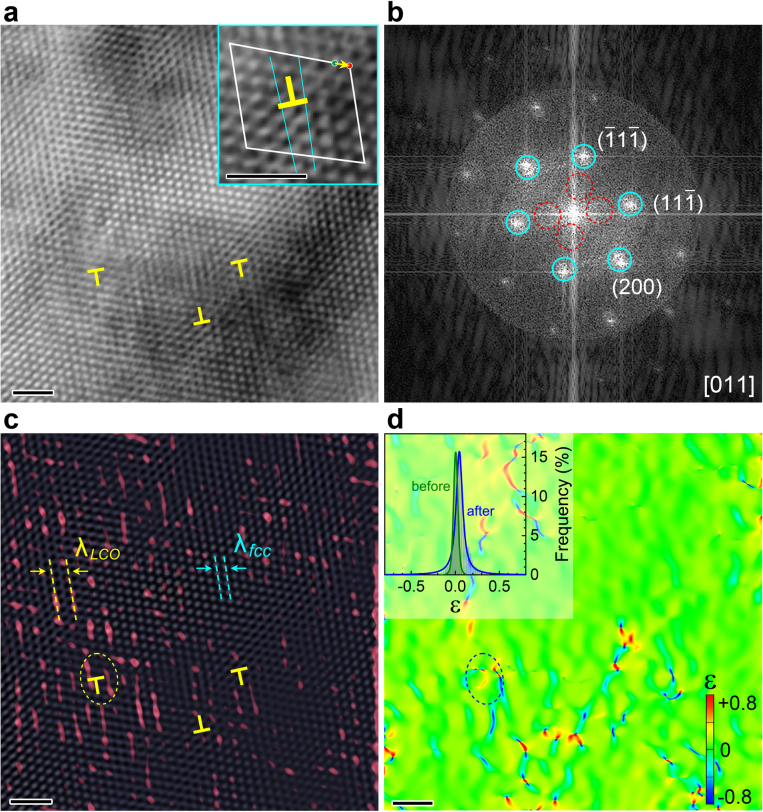
Interaction between dislocations and LCO regions by means of the lattice-image-based geometric phase analysis (GPA) mapping. (**a**) Lattice image of fcc crystal. Several dislocations are labeled by yellow Τs. Zone axis: [011]. Inset: Burgers circuit enclosing one full dislocation in yellow, with the arrow indicating the Burgers vector from the starting point to ending point. (**b**) Indexed Fourier transform pattern. Blue circles: fcc diffraction spots. Red circles: extra superlattice scattering by LCO regions. (**c**) Overlay of two inverse Fourier transform images. Dash circle encloses one LCO regions, along with the extra half plane of one dislocation. Azury lattice: fcc matrix. Red spots: LCO regions. *λ*_*LCO*_ and *λ*_*fcc*_: inter-planar spacing of (111) planes for LCO regions and fcc lattice. (**d**) Geometric phase analysis (GPA) imaging of (**a**) Yellow areas: strain contrast induced by LCO regions. Two connected slender areas in blue and red: dislocation. Scale bar of lattice strain (ε): 0.8 (tensile) to -0.8 (compressive). Scale bar in (**a, c, d**): 1 nm. Inset: distribution of full-field strains, *ε*, before and after tensile deformation.

HDI hardening is indispensable due to the HDI-hardening rate accounting for over 50% of the global hardening rate (Fig. 5, bottom). The local HDI stress causes the HDI hardening by GNDs (Supplementary Note 7)^39^. HDI stress is produced when a specific GND pattern, for example, a pile up and a group of loops, is blocked by either precipitates or grain boundaries^39,42–44^. However, small UFGs make these patterns of GNDs difficult to form. Meanwhile, the dislocations of mass multiplication rapidly evolve into tangles, usually having a negligible HDI stress^39^. Here we propose the LCO regions as the microstructural source for HDI stress. On one hand, the LCO regions are mechanically stable, rather than be destroyed by gliding dislocations, showing the almost unchangeable size and volume fraction before and after tensile deformation (Extended Data Fig. 1d5). On the other hand, the strain field is attached to an ordered LCO region (Fig. 1d), producing the strain-field interaction with gliding dislocations^43^. This scenario is consistent with that in an ordered precipitate of intermetallic phase, inducing the strain-field interaction with dislocations^45–48^. Conventionally, shearing is assumed as the interaction of LCO regions with gliding dislocations due to their tiny sizes. However, this incurs a problem that shearing cannot induce HDI stress, whereas bypassing can due to the constantly produced dislocation loops^43,44^. We propose that the HDI stress is induced by the strain-field interaction between dislocations and LCO region, or the cluster of LCO regions as a forest. To verify this (Supplementary Note 8), we conducted high-resolution TEM observations in fcc grains, showing several edge dislocations (extra half-planes labelled by yellow ‘Τ’ symbols) and LCO regions as evidenced by their characteristic electron diffraction^43^ (Extended Data Fig. 4a,b). The LCO regions (red spots) distribute on the fcc lattice (azury spots) in the overlay of two inverse Fourier transform images (Extended Data Fig. 4c). One edge dislocation (yellow-coloured Τ) inserts into the middle of the circled LCO region. The corresponding GPA map shows the coupling of the strain field between the LCO region and dislocations (Extended Data Fig. 4d). The average strain is 0.2% before tensile deformation, whereas it markedly rises to 6.9% after plastic deformation (Extended Data Fig. 4d, inset). The strain-field interaction induces an increased local strain near the LCO region. This indicates a trapping effect on moving dislocations when migrating through the strain field of LCO regions. By the definition of GND^33^, these dislocations that interact with LCO regions are GNDs compensating for local strain incompatibility. At this moment, an extra force, that is, HDI stress, is needed such that GNDs are de-trapped from the strain field and continue to move. The strain field, especially induced by the forest of LCO regions, will increase opportunities for GNDs to interact with each other. Therefore, extra HDI hardening is expected by the LCO regions.

In summary, here we show to exploit and stabilize premature necking during LB propagation to induce combined work hardening for ductility in MPEAs with UHYSs. These results help to understand and design the well-balanced strength and ductility and are easily transferable to other MPEAs containing the LCO regions as intrinsic heterogeneities.

## Methods

### Materials, processing and heat treatment

Nickel, cobalt and vanadium (all of them, >99.9% purity) were selected for the VCoNi alloy smelting. The ingot was cast into the mould of 130 mm diameter under an argon atmosphere by using the arc-melting technique. The ingot was turned upside down and then re-melted five times for chemical homogeneity. The ingot was hot forged at 1,423 K to the plate with a dimension of 12 × 100 × 600 mm^3^, homogenization treated in a vacuum at 1,373 K for 2 h, followed by quenching in water. Cold rolling was finally conducted with a 90% thickness reduction to obtain thin sheets of 1 mm thickness. Recrystallization annealing was conducted at 1,173 K for 150 s. The chemical composition was the equiatomic 33V–34Co–33Ni (atomic percentage) in this ternary VCoNi MPEA.

### Microstructural characterization

#### XRD

XRD study was performed to obtain detailed information on the phase structure using a Rigaku SmartLab 9 X-ray diffractometer with Cu Kα radiation (*λ* = 1.5406 Å) at 35 kV. The mechanically polished samples were scanned through the 2*θ* range from 30.00° to 100.00° with a step size of 0.02° and counting time of 2 s. The volume fraction of L1_2_ plates was determined by the relative integrated intensities of the characteristic diffraction peaks as follows^49^:

1 $${f}_{{\rm{L}}{1}_{2}}=\frac{\sum {{{I}}}_{{\rm{L}}{1}_{2}}}{\sum {{{I}}}_{{\rm{fcc}}}+\sum {{{I}}}_{{\rm{L}}{1}_{2}}},$$

where $\sum {{{I}}}_{{\rm{fcc}}}$ and $\sum {{{I}}}_{{\rm{L}}{1}_{2}}$ are the sum of the integrated intensities of the characteristic diffraction peaks, that is, (111), (200), (220) and (311) for fcc and L1_2_, respectively.

#### SEM EBSD observation

The microstructural observation was conducted by using a high-resolution field-emission ZEISS GeminiSEM 300 scanning electron microscope (SEM) equipped with a fully automatic Oxford Instruments Aztec 2.0 electron backscatter diffraction (EBSD) system (Channel 5 software), with a scanning step of 40 nm during the EBSD acquisition. The high-angle grain boundaries were defined with a misorientation angle larger than 15°. The statistical mean grain size was obtained by measuring the original fcc grains, without the effect of subdivision by L1_2_ plates inside the grain interiors.

#### TEM observation

Thin foils for TEM observations were mechanically polished to ~50 μm thickness and then punched to discs of 3 mm in diameter. The foils were perforated by twin-jet electron polishing with a solution of 20 vol% perchloric acid and 80 vol% acetic acid at –25 °C and a voltage of 50 mA. Atomic-resolution HAADF images were performed in an aberration-corrected scanning transmission electron microscope (FEI Titan Cubed Themis G2 300) operated at 300 kV, equipped with Super-X energy-dispersive X-ray spectroscopy with four window-less silicon-drift detectors. The thickness is around 30 nm in areas of TEM foils for the imaging of various LCO regions, especially energy-dispersive X-ray spectroscopy mapping.

The weak-beam TEM dark-field imaging was conducted to accurately determine the dislocation lines and their patterns. A weak beam refers to imaging under the diffraction contrast with reflection **g** set far off the Bragg condition. This weak-beam condition may effectively reduce the interference effects caused by the specimen thickness and dynamic scattering on the contrast of dislocation lines^50,51^. The bright contrast of dislocations emerges from an otherwise faint background. Here the **g**/3**g** condition, with **g** = 11$\bar{1}$, has been employed to generate weak-beam dark-field images in fcc grains, which are recorded in an FEI Tecnai G2 F20 TEM instrument operated at 200 kV and an FEI Titan Cubed Themis G2 300 instrument operated at 300 kV.

The average size and spacing of LCO regions were measured in the energy-filtered dark-field TEM images and simultaneously in at least 20 inverse fast Fourier transform atomic-resolution TEM images. With the assumption of a uniform distribution of tiny LCO regions in three-dimensional space, the volume fraction (*f*_V_) of LCO regions is calculated as follows^52,53^:

2 $${f}_{{\rm{V}}}=\frac{\uppi }{4}\times \frac{1}{{\left[\frac{\lambda }{d\sqrt{2/3}}+1\right]}^{2}},$$

where *d* is the average size of the LCO regions and *λ* is the average interspacing.

#### Focused-ion-beam method

Thin TEM foils at the site-specific LB front were fabricated by liftouts and cross-section milling in an FEI Helios Nanolab G3 CX focused-ion-beam SEM device. The area of interest was selected and protected by platinum deposition. The initial milling was executed at a high ion-beam current (that is, ion beam parallel to the *y* axis), forming a film that was 10 μm wide (*x* direction), 10 μm high (*y* direction) and 2 μm thick (*z* direction). Then, this film was removed from the milled trench by using a micromanipulator and placed on a Cu grid. The film was further thinned gradually to avoid damaging by decreasing the ion-beam currents from 0.79 nA to 40 pA at a voltage of 30 kV until the thickness was less than 80 nm, which is suitable for TEM observations. The surface was finally cleaned to remove the damage of the ion beam at a relatively small voltage.

#### High-resolution TEM lattice-image-based GPA mapping

The GPA map shows the strain-field contours of the LCO regions on the basis of the change in lattice fringe spacing across the whole lattice image. The Fourier transform pattern related to different crystal planes (*hkl*) was obtained at first based on high-resolution lattice images with a specific zone axis. A perfect crystal lattice gives rise to sharply peaked frequency components, whereas the Bragg reflections broaden due to local lattice distortions. The VCoNi solution is a mixture of V, Co and Ni atoms, with an evidently differing atomic radius. In the normal direction of the close-packed ($11\bar{1}$) plane, tensile and compressive strain will be caused due to the local enrichment of larger V and smaller Co/Ni atoms, respectively. We placed a circular Gaussian mask on the reflection of ($11\bar{1}$) to obtain the strain map of close-packed planes^20^. The resolution was set at 0.25 nm to ensure the full display of lattice strain caused by LCO regions.

### Mechanical tests

#### Uniaxial tensile test at room temperature

For the uniaxial tensile test, unload–reload test and stress-relaxation test, all the samples were dog-bone shaped, with a gauge section of 5 × 1 mm^2^ and 23 mm in length. The samples were cut from thin sheets of 1 mm thickness along the rolling direction. All the tensile tests at room temperature (298 K) were performed in an Instron 5967 machine at a strain rate of 5 × 10^−4^ s^−1^. The contacted extensometer was used to measure strains during tensile loading.

#### Tensile test at cryogenic temperatures

Tensile tests were conducted at a strain rate of 5 × 10^−4^ s^−1^ at both 77 and 4 K in a displacement-controlled mode in an electronic–mechanical test machine (MTS model SANS UTM 5305S, with a load capacity of 300 kN). A cryogenic-grade extensometer (Epsilon model 3542-010M-LT, with a gauge length of 10 mm) was used to record the full-range deformation. In particular, tensile tests at 4 K were conducted by immersing the whole specimen and test jigs into liquid helium in a home-made cryostat installed on the cross-beam of the test machine. All the interior components of the sample chamber were insulated by a high-purity copper radiation shield and multilayer insulation material, which effectively prevented liquid helium from evaporating. The liquid-helium level was monitored with a cryogenic liquid-level meter (Cryomagnetics model LM-500) and the liquid helium was refilled to maintain the defined level during the tensile process. A thermometer was installed on the inner wall of the sample chamber and the height of the thermometer was consistent with the tested sample, which was used not only to obtain accurate temperature but also to control the temperature inside the sample chamber.

#### Unload–reload tensile test

The testing was conducted to measure the HDI stress. The stretched specimen was subjected to successive cycles of unloading and reloading at several strains. The mechanical hysteresis loops appeared if GND-based heterodeformation happens. The HDI stress is calculated as follows^54^:

3 $${\sigma }_{{{\rm{HDI}}}}=\frac{\left({\sigma }_{{\rm{u}}}+{\sigma }_{{\rm{r}}}\right)}{2},$$

where *σ*_u_ and *σ*_r_ are the yield stresses on the unload and reload cycles, respectively, which were obtained from the hysteresis loops during each unload–reload cycle.

#### Synchrotron-based high-energy XRD in situ tensile test

The in situ tests were performed at the 11-ID-C beamline, Advanced Photon Source, Argonne National Laboratory. A monochromatic X-ray beam was used with an energy of 71.676 keV at a wavelength of 0.1173 Å. The beam size was 500 μm (along the width direction of the tensile sample) × 100 μm (along the longitudinal direction). The tensile tests were performed at room temperature and at a strain rate of 1 × 10^−3^ s^−1^. The gauge section was 10.0 (length) × 3.0 (width) × 0.5 (thickness) mm^3^. The engineering strain was measured from the cross-head displacement and corrected on the basis of the elasticity modulus. During tensile deformation, the scattering intensity was collected using a two-dimensional detector placed 1,770 mm from the tested sample. The two-dimensional diffraction data were transformed into a one-dimensional line profile by integrating over a ±5° azimuth range around the longitudinal direction and transversal direction of the specimen with GSAS-II software^55^. The crystallographic planes were determined from the diffraction patterns and lattice strains, *ε*_*hkl*_, were calculated by the change in the corresponding interplanar spacing relative to the lattice spacing in the undeformed state as follows:

4 $${\varepsilon }_{{hkl}}=\frac{({d}_{{hkl}}-{d}_{{hkl}}^{0})}{{d}_{{hkl}}^{0}},$$

where ${d}_{{hkl}}^{0}$ is the *d* spacing of the (*hkl*) grain family at zero applied stress. The full-width at half-maximum of the physical lineshape for the tested sample was calibrated by extracting the instrumental broadening from the measured broadening as follows:

5 $${\rm{FWHM}}=\left[{\left({{\rm{FWHM}}}_{{{\rm{measured}}}}\right)}^{2}-{\left({{\rm{FWHM}}}_{{{\rm{instrumental}}}}\right)}^{2}\right].$$

The instrumental broadening was characterized using a near-perfect (broadening-free) CeO_2_ powder.

The dislocation density (*ρ*) was calculated from the synchrotron XRD profiles by using the modified William–Hall method^56^, assuming that the broadening of diffraction peaks is linked to the microstructural parameters including the average grain size (*D*) and *ρ*:

6 $$\Delta K\cong \frac{0.9}{D}+{\left(\frac{\uppi {A}^{2}{b}^{2}}{2}\right)}^{\frac{1}{2}}{\rho }^{\frac{1}{2}}\left(K{C}^{1/2}\right),$$

where

7 $$K=\frac{2\sin \theta }{\lambda }$$

and

8 $$\Delta {\rm{K}}=\frac{\cos \theta (\Delta 2\theta )}{\lambda }.$$

Also, *θ* is the diffraction angle at the exact Bragg position; *λ*, the X-ray wavelength; Δ2*θ* is the full-width at half-maximum of the diffraction peak at *θ*; *A* is a constant determined by the effective outer cutoff radius of dislocations (here we used *A* = 1 in the present study); *b* is the magnitude of Burgers vector (0.255 nm); and *C* is the average dislocation contrast factor^57^. For each (*hkl*) reflection, the value of *C* can be determined as

9 $$C={C}_{H00}\left\{1-q\left[\frac{{h}^{2}{k}^{2}+{k}^{2}{l}^{\;2}+{h}^{2}{l}^{\;2}}{{\left({h}^{2}+{k}^{2}+{l}^{\;2}\right)}^{2}}\right]\right\},$$

where *C*_*H*00_ and *q* are constants related to the anisotropic elastic constants (*C*_11_, *C*_12_ and *C*_44_) of the tested alloy. Also, *C*_*H*00_ and *q* depend on the elastic anisotropy *A*_*i*_ = 2*C*_44_(*C*_11_ – *C*_12_) and the ratio *C*_12_/*C*_44_.

10 $${C}_{H00}=a\left[1-\exp \left(-{A}_{i}/b\right)\right]+c{A}_{i}+d$$

11 $$q=a\left[1-\exp \left(-{A}_{i}/b\right)\right]+c{A}_{i}+d$$

For the present VCoNi alloy, elastic constants *C*_11_, *C*_12_ and *C*_44_ equal to 169, 82 and 96 GPa. The constants for the two groups, namely, *a*, *b*, *c* and *d*, are (0.1607, 1.8610, 0.0196 and 0.0928) and (4.6080, 0.8706, 0.0924 and –3.2550), and are used for the calculation of *C*_*H*00_ and *q*, respectively.

#### Stress-relaxation tensile testing

The aim of this test was to figure out the density evolution of mobile dislocations during tensile deformation. The specimen was first stretched at a strain rate of 5 × 10^−4^ s^−1^ to certain strain, whereas the cross-beam head was held to allow stress relaxation for 180 s. Then, this relaxed specimen was reloaded to the same stress level as before at a strain rate of 10^−1^ s^−1^ to conduct the second cycle of relaxation. In total, four cycles of repeated stress relaxation were carried out at each designed strain. The mobile dislocation density, ${\rho }_{\mathrm{m}}/{\rho }_{\mathrm{m}_{0}}$, exhibits an empirical power law with time (*t*) (ref. ^36^):

12 $$\frac{{\rho }_{\mathrm{m}}}{{\rho }_{\mathrm{m}_{0}}}={\left(\frac{{c}_{{\rm{r}}}}{t+{c}_{{\rm{r}}}}\right)}^{\frac{\beta }{1+\beta }},$$

where ${\rho }_{\mathrm{m}_{0}}$ is the dislocation density at the onset of each transient, *c*_*r*_ is a time constant and *β* is a dimensionless immobilization parameter. The derivation of *c*_r_ and *β* is described below.

A single stress-relaxation transient exhibits a logarithmic variation of stress drop with time elapsed. The apparent activation volume *V*_a_ is determined by fitting the logarithmic stress-relaxation curve as

13 $$\Delta \tau (t)=-\frac{{k}_{{\rm{B}}}T}{{V}_{{\rm{a}}}}\,\mathrm{ln}\left(1+\frac{t}{{C}_{{\rm{r}}}}\right),$$

where Δ*τ*(*t*) is the stress drop at time *t*, *T* is the temperature (Kelvin) and *k*_B_ is the Boltzmann constant; consequently, time constant *C*_r_ and apparent activation volume *V*_a_ are obtained. The physical activation volume V* is determined as

14 $${V}^{\,\ast }={k}_{{\rm{B}}}T\frac{\mathrm{ln}({\dot{\gamma }}_{i2}/{\dot{\gamma }}_{f\;1})}{\Delta {\tau }^{\,\ast }},$$

where ${\dot{\gamma }}_{i2}$ and ${\dot{\gamma }}_{f\;1}$ are the shear strain rate at the onset of relaxation 2 and the end of relaxation 1, respectively. Δτ* is the change in thermal component under relaxation condition:

15 $$\Delta {\tau }^{\ast }=(1+K/M\,)\Delta \tau,$$

where *K* can be approximated by the strain-hardening rate of the monotonic tensile curve, and *M* is the stiffness of the specimen−machine assembly. Additionally, the dimensionless immobilization parameter *β* can be determined as

16 $$\beta =\frac{\varOmega }{1+K/M}-1,$$

where parameter *Ω* can be expressed as

17 $$\varOmega =\frac{{V}_{{\rm{a}}}}{{V}^{\,\ast}}.$$

Finally, the density of mobile dislocations after relaxation can be obtained.

#### DIC-based in situ tensile test

This test was used to quantitatively measure the distribution of full-field strain during tensile deformation. The spot pattern was sprayed onto a white background on the specimen surface. The displacement of the pattern was tracked using a 1.2 megapixel charge-coupled device camera with a resolution of 2,848 × 2,848 pixels^2^ at 1 frame s^−1^. The recorded camera images were then analysed for local strains using ARAMIS v. 6.1 software. The strain contours were calculated with a spatial resolution of 275 nm pixel^–1^.

The strain gradient *λ* induced by the heterogeneous strain distribution is calculated as

18 $$\lambda =\frac{\partial \varepsilon }{\partial x},$$

where *ε* is the longitudinal strain measured by the DIC tensile testing and *x* is the distance from the LB front. Since the GNDs play a pivotal role in accommodating the local strain incompatibility, a classical method from the strain-gradient theory^37,38^ is used to estimate the GND density (*ρ*_GND_) as follows:

19 $${\rho }_{{{\rm{GND}}}}=\frac{\lambda }{b},$$

where *b* is the magnitude of Burgers vector (0.255 nm).

### Calculation of stress triaxiality by FEM simulation

The FEM calculation and simulation were carried out by means of the Abaqus/Standard code. The three-dimensional model was created according to the actual dimensions of the tensile specimen, and the simulated specimen was loaded in a similar way to real tensile testing. During the simulated testing, the upper grip of the model was fixed, whereas the lower grip was only allowed to move along the axial direction. The model was loaded on the lower-end surface to simulate the displacement-controlled tensile test. The analysis was set up in the large-deformation mode, static three-dimensional calculation with default stabilizing options and 41,124 hexahedral elements, type C3D20R (ref. ^31^). The reaction force on the lower-end surface and the elongation of gauge section were used to calculate the engineering stress and strain. A trigger element with a slightly lower strength of 75 MPa than all the other elements was severed as the starting point for LB propagation^58^. The simulation was regarded as reliable until the following targets are achieved: (a) the simulated engineering stress–strain relation achieved the best agreement with the experimental one; (b) the LB formation, propagation and subsequent uniform deformation were precisely reproduced.

The triaxiality parameter^32^ *η* is defined as the hydrostatic press (*σ*_H_) divided by von Mises stress (*σ*_M_):

20 $$\eta =\frac{{\sigma }_{{\rm{H}}}}{{\sigma }_{{\rm{M}}}},$$

21 $${\sigma }_{{\rm{H}}}=\frac{{\sigma }_{1}+{\sigma }_{2}+{\sigma }_{3}}{3},$$

22 $${\sigma }_{{\rm{M}}}=\sqrt{\frac{1}{2}\left[{\left({\sigma }_{1}-{\sigma }_{2}\right)}^{2}{+\left({\sigma }_{2}-{\sigma }_{3}\right)}^{2}+{\left({\sigma }_{3}-{\sigma }_{1}\right)}^{2}\right]},$$

where *σ*_1_, *σ*_2_ and *σ*_3_ are the principal stress values along the three stress directions.

## Online content

Any methods, additional references, Nature Portfolio reporting summaries, source data, extended data, supplementary information, acknowledgements, peer review information; details of author contributions and competing interests; and statements of data and code availability are available at 10.1038/s41563-024-01871-7.

### Supplementary information

Supplementary Information Supplementary Notes 1–8 and Figs. 1–6.

## Data Availability

All data that support the findings of this study are reported in the Article and its Supplementary Information.

## References

[1] Lu K (2010). The future of metals. Science.

[2] Ritchie RO (2011). The conflicts between strength and toughness. Nat. Mater..

[3] Li ZM, Pradeep KG, Deng Y, Raabe D, Tasan CC (2016). Metastable high-entropy dual-phase alloys overcome the strength-ductility trade-off. Nature.

[4] Ma E, Wu XL (2019). Tailoring heterogeneities in high-entropy alloys to promote strength-ductility synergy. Nat. Commun..

[5] Yang Y (2021). Bifunctional nanoprecipitates strengthen and ductilize a medium-entropy alloy. Nature.

[6] Lu L, Chen X, Huang X, Lu K (2009). Revealing the maximum strength in nanotwinned copper. Science.

[7] Wu XL (2015). Heterogeneous lamella structure unites ultrafine-grain strength with coarse-grain ductility. Proc. Natl Acad. Sci. USA.

[8] He BB (2017). High dislocation density-induced large ductility in deformed and partitioned steels. Science.

[9] Li YJ (2023). Ductile 2-GPa steels with hierarchical substructure. Science.

[10] Jiang SH (2017). Ultrastrong steel via minimal lattice misfit and high-density nanoprecipitation. Nature.

[11] Du XH (2020). Dual heterogeneous structures lead to ultrahigh strength and uniform ductility in a Co-Cr-Ni medium-entropy alloy. Nat. Commun..

[12] Chung H (2023). Doubled strength and ductility via maraging effect and dynamic precipitate transformation in ultrastrong medium-entropy alloy. Nat. Commun..

[13] Kwon H (2023). High-density nanoprecipitates and phase reversion via maraging enable ultrastrong yet strain-hardenable medium-entropy alloy. Acta Mater..

[14] Liu CT, Stiegler JO (1984). Ductile ordered intermetallic alloys. Science.

[15] Sohn SS (2019). Ultrastrong medium-entropy single-phase alloys designed via severe lattice distortion. Adv. Mater..

[16] Park JM (2021). Ultra-strong and strain-hardenable ultrafine-grained medium-entropy alloy via enhanced grain-boundary strengthening. Mater. Res. Lett..

[17] Li QJ, Sheng H, Ma E (2019). Strengthening in multi-principal element alloys with local-chemical-order roughened dislocation pathways. Nat. Commun..

[18] Wang L (2023). Tailoring planar slip to achieve pure metal-like ductility in body-centred-cubic multi-principal element alloys. Nat. Mater..

[19] Zhang R (2020). Short-range order and its impact on the CrCoNi medium-entropy alloy. Nature.

[20] Chen XF (2021). Direct observation of chemical short-range order in a medium-entropy alloy. Nature.

[21] Wu XL (2023). Chemical short-range orders in high-/medium-entropy alloys. J. Mater. Sci. Technol..

[22] Wang J, Jiang P, Yuan FP, Wu XL (2022). Chemical medium-range order in a medium-entropy alloy. Nat. Commun..

[23] Order or disorder. *Nat. Mater*. **22**, 925 (2023). 10.1038/s41563-023-01636-8 37524823

[24] Hÿtch MJ, Snoeck E, Kilaas R (1998). Quantitative measurement of displacement and strain fields from HREM micrographs. Ultramicroscopy.

[25] Gludovatz B (2014). A fracture-resistant high-entropy alloy for cryogenic applications. Science.

[26] Liu XR (2022). Mechanical property comparisons between CrCoNi medium-entropy alloy and 316 stainless steels. J. Mater. Sci. Technol..

[27] Edalati K, Furuta T, Daio T, Kuramoto S, Horita Z (2015). High strength and high uniform ductility in a severely deformed iron alloy by lattice softening and multimodal-structure formation. Mater. Res. Lett..

[28] Fan L (2020). Ultrahigh strength and ductility in newly developed materials with coherent nanolamellar architectures. Nat. Commun..

[29] Liang YJ (2018). High-content ductile coherent nanoprecipitates achieve ultrastrong high-entropy alloys. Nat. Commun..

[30] Reddy SR (2019). Nanostructuring with structural-compositional dual heterogeneities enhances strength-ductility synergy in eutectic high entropy alloy. Sci. Rep..

[31] Schwab R, Ruff V (2013). On the nature of the yield point phenomenon. Acta Mater..

[32] Hancock JW, Mackenzie AC (1976). On the mechanisms of ductile failure in high-strength steels subjected to multi-axial stress-states. J. Mech. Phys. Solids.

[33] Ashby MF (1970). The deformation of plastically non-homogeneous materials. Philos. Mag..

[34] Wilson DV (1965). Reversible work hardening in alloys of cubic metals. Acta Metall..

[35] Hansen N, Huang X (1998). Microstructure and flow stress of polycrystals and single crystals. Acta Mater..

[36] Caillard D, Martin JL (2003). Thermally Activated Mechanisms in Crystal Plasticity.

[37] Gao H, Huang Y, Nix WD, Hutchinson JW (1999). Mechanism-based strain gradient plasticity—I. Theory. J. Mech. Phys. Solids.

[38] Li XY, Lu L, Li JG, Zhang X, Gao HJ (2020). Mechanical properties and deformation mechanisms of gradient nanostructured metals and alloys. Nat. Rev. Mater..

[39] Zhu YT, Wu XL (2023). Heterostructured materials. Prog. Mater. Sci..

[40] Chen Z (2022). Unraveling the origin of extra strengthening in gradient nanotwinned metals. Proc. Natl Acad. Sci. USA.

[41] Naeem M (2020). Cooperative deformation in high-entropy alloys at ultralow temperatures. Sci. Adv..

[42] Xiang Y, Vlassak JJ (2005). Bauschinger effect in thin metal films. Scr. Mater..

[43] Stoltz RE, Pelloux RM (1976). The Bauschinger effect in precipitation strengthened aluminum alloys. Metall. Trans. A.

[44] Brown LM, Clarke DR (1975). Work hardening due to internal stresses in composite materials. Acta Metall..

[45] Hazzledine PM, Sun YQ (1992). The strain field and work-hardening from antiphase boundary tubes in ordered alloys. Mater. Sci. Eng. A.

[46] Lin HR, Hendrickson AA (1988). The prediction of precipitation strengthening in microalloyed steels. Metall. Trans. A.

[47] Huther W, Reppich B (1978). Interaction of dislocations with coherent, stress-free, ordered particles. Z. Metallkde..

[48] Le Fournier M, Douin J, Gatel C, Pettinari-Sturmel F, Donnadieu P (2012). Measurement and modeling of the elastic strain around nanoprecipitates. Rev. Metall. Cah. Inf. Tech..

[49] Cheng S (2010). An assessment of the contributing factors to the superior properties of a nanostructured steel using in situ high-energy X-ray diffraction. Acta Mater..

[50] Cockayne DJH, Ray ILF, Whelan MJ (1969). Investigations of dislocation strain fields using weak beams. Philos. Mag..

[51] Veyssière P (2006). The weak-beam technique applied to the analysis of materials properties. J. Mater. Sci..

[52] Brown LM, Ham RK (1971). Strengthening Methods in Crystals.

[53] Fuller CB, Seidman DN, Dunand DC (2003). Mechanical properties of Al (Sc, Zr) alloys at ambient and elevated temperatures. Acta Mater..

[54] Yang MX, Pan Y, Yuan FP, Zhu YT, Wu XL (2016). Back stress strengthening and strain hardening in gradient structure. Mater. Res. Lett..

[55] Larson, A. C. & Von Dreele, R. B. *General Structure Analysis System (GSAS)*. Report No. LAUR 86-748 (Los Alamos National Laboratory, 2004).

[56] Ungár T, Ott S, Sanders PG, Borbély A, Weertman JR (1998). Dislocations, grain size and planar faults in nanostructured copper determined by high resolution X-ray diffraction and a new procedure of peak profile analysis. Acta Mater..

[57] Ungár T, Dragomir I, Révész Á, Borbély A (1999). The contrast factors of dislocations in cubic crystals: the dislocation model of strain anisotropy in practice. J. Appl. Cryst..

[58] Hallai JF, Kyriakides S (2013). Underlying material response for Lüders-like instabilities. Int. J. Plast..

