# Supplementary material for: Harnessing instability for work hardening in multi-principal element alloys
Source: Nat Mater. 2024 Apr 11;23(6):755–61. doi: 10.1038/s41563-024-01871-7 (PMC11150159; doi:10.1038/s41563-024-01871-7)
Supplement: Supplementary file 1 — Supplementary Notes 1–8 and Figs. 1–6. [file 41563_2024_1871_MOESM1_ESM.pdf]

nature materials

Article

https://doi.org/10.1038/s41563-024-01871-7

Harnessing instability for work hardening in multi-principal element alloys

In the format provided by the authors and unedited

Contents

Notes

Note 1. Atom occupation of elemental species in the ordered $L1_2$ plate..2

Note 2. Presence of hcp structure in the fcc-$L1_2$ plate 2

Note 3. Origin of yield drop and LB initiation 2

Note 4. Effect of v_{max} 2

Note 5. Phase transformation..2

Note 6. Premature necking and work hardening in M_7..2

Note 7. Strain gradient and GNDs..3

Note 8. Strain-field interaction 3

Figures

Fig. 1. Atom occupation of elemental species in the ordered $L1_2$ plate measured by the EDS mapping (corresponding to the HAADF lattice image in Fig. 1b), showing the presence of a small quantity of hcp structure in fcc lattice..4

Fig. 2. Two-dimensional full-field strain measurements during tensile deformation in two microstructures of differing yield strengths by the digital-image-correlation (DIC) in situ tensile testing................5

Fig. 3. FEM calculations and simulations of tensile deformation in various microstructures.................7

Fig. 4. Measurement of mobile dislocation density in the microstructure (Fig. 1a) of yield strength of 2 GPa (Fig. 2a) by the stress relaxation tensile testing..9

Fig. 5. Dislocation production at the site-specific locations during the LB propagation by TEM observations..10

Fig. 6. Measurement of HDI stress (σ_{HDI}) by the unload-reload tensile testing at room temperature in the M_4 microstructure of yield strength of 2 GPa (Fig. 2a)..11

References..12

Note 1. Atom occupation of elemental species in the ordered $L1_2$ plate

The corresponding energy-dispersive X-ray spectroscopy (EDS) mapping in Supplementary Fig. 1b shows that V atoms and Co/Ni atoms are situated in the alternate (200) planes as the characteristic occupation of element species in the fcc-$L1_2$ lattice.

Note 2. Presence of hcp structure in the fcc-$L1_2$ plate

A minor amount of close-packed hexagonal (hcp) crystal structure is observed in the $L1_2$ lamellae in Fig. 1b. Their formation is much likely due to the local composition fluctuation [1,2]. Yet, the hcp layer of usually a few atomic layer thick is too thin to obtain the decisive diffraction pattern.

Note 3. Origin of yield drop and LB initiation

Yield-drop and LB initiation are usually ascribed to the initial dislocation lack and resultant low work hardening ability [3-6]. This is indeed true even in the present UHYS UFGs as shown by the following dislocation density results.

Note 4. Effect of v_{max}

The stable v_{max} plateau during the LB propagation gives two hints. One is the effective restraint of premature necking during the LB propagation, while the other is work hardening produced soon after the onset of LB initiation. Otherwise, this early necking will develop due to such a large v_{max}.

Note 5. Phase transformation

Neither phase transformation nor deformation twinning is observed during room and cryogenic deformation as detected by both XRD spectra and TEM observations (Extended data Figs. 1,2,3).

Note 6. Premature necking and work hardening in M_7

We also conducted the concurrent study of LB propagation and dislocation behavior in a micron-grained microstructure (MG), called M_7 (Extended data Figs. 1a,1e), for comparison with the results in UFG M_4. M_7 has an increased $\bar{d}$ of 1.26 μm and decreased σ_y of 1.6 GPa. On the instability tendency, the initial v_{max} peak drops to 0.45 in M_7, only half that in M_4 (Fig. 3a), while the v_{max} plateau during the LB propagation keeps almost unchangeable compared to that in M_4. On the dislocation density, a low initial density appears in M_4 (Extended data Fig. 2b3), similar to that in M_7. Δρ during the LB propagation decreases from 12×10^{14} m^{-2} in M_4 to only 3.9×10^{14} m^{-2} in M_7 (Extended data Fig. 2b3), along with a decreased speed of dislocation multiplication at the LB front from 4.6×10^{13} m^{-2} s^{-1} to 1.9×10^{13} m^{-2} s^{-1}. This is the reason of decreased LB strains. By contrast, Δρ during the entire tensile deformation increases from 17×10^{14} m^{-2} in M_4 to 21.7 $\times10^{14}$ m^{-2} in M_7, leading to an enhanced uniform strains in M_7. Meanwhile, both η_{max} and λ_{max} decreases as a result of a decreased v_{max} (Supplementary Figs. 3b3,2b4). All these changes are ascribed to the increased $\bar{d}$ in M_7. Namely, the bigger $\bar{d}$, the less tendency of premature instability will be. Yet, the premature necking happens still in M_7 due to the larger v_{max} than that during uniform defor-mation. Further, the yield-drop followed by LB appears in the stress-strain curve of M_7 of less σ_y (Extended data Fig.

1a), similar to that happened in M_4 of higher σ_y. The primary cause is the same, i.e., the initial shortage of mobile dislocations and low work hardening ability. The premature necking doubtlessly supports the conclusion.

Note 7. Strain gradient and GNDs

GNDs are produced to accommodate strain gradient. These GNDs have two roles. One is to relieve plastic incompatibility at the LB front, while the other is to offer extra HDI hardening.

Note 8. Strain-field interaction

The lattice image-based GPA mapping shows the strain field interaction of gliding dislocations with LCO regions (Extended data Fig. 4c). As to the LCO region circled, the extra half plane of one dislocation (yellow T) happens to be inserted into the middle of two $(\bar{1}1\bar{1})$ planes in this LCO region. This indicates that the dislocation is cutting through the LCO region. In the corresponding GPA mapping (Extended data Fig. 4d), the strain field contrast appears by both LCO regions and edge dislocations. The strain field of an edge dislocation is the joined slender area by compressive and tensile strain field in blue and red below and above the slip plane. The overlay of strain fields of two kinds is visible, indicating the strain field interaction. Inset shows the full-field strain distribution measured based on the GPA mapping. The average strain is 0.2% before tensile deformation, while it markedly rises to 6.9% after plastic deformation. The interaction of LCO regions with gliding dislocations will cause a local, extra HDI stress, which exerts on the dislocations when they approach. This entails a trapping effect on the moving dislocations. As a result, the dislocation line migrating through the field of LCO regions slows down, and its forward progression has to proceed via local segments cutting through and de-trapping from the LCO regions [2]. The strain-field interaction facilitates the LCO regions to trap and pin gliding dislocations (Extended data Figs. 4c,d).

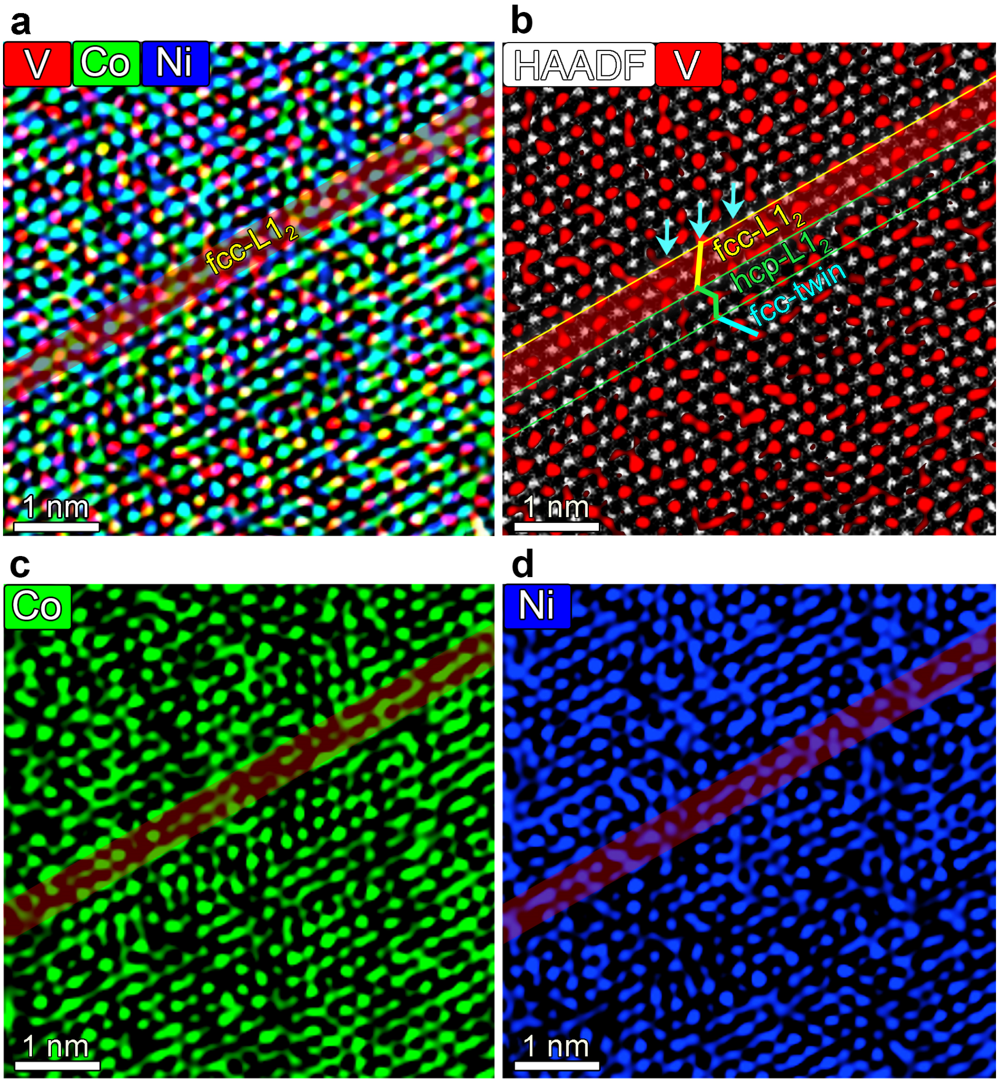

Supplementary Fig. 1 | Atom occupation of elemental species in the ordered $\mathrm{L1_2}$ plate measured by the EDS mapping (corresponding to the HAADF lattice image in Fig. 1b), showing the presence of a small quantity of hcp structure in fcc lattice. (**a**) EDS map of three elements (V, Co, and Ni). Each spot is actually one atom column of ~30 nm thick, i.e., the thickness in areas of TEM foils for EDS measurements. Four maps indicate the co-existence of V, Co, and Ni in each column, just with different proportion for each element. One plate (in red) in for maps indicates the fcc-$\mathrm{L1_2}$ of 2 atom-layer thick. (**b**) Overlay of HAADF image and V map (V atoms are colored in red). The atom ordering in a typical $\mathrm{A_3B}$-like (here B is V atom) fcc $\mathrm{L1_2}$ features an alternative layered sequence of V (a few are labeled by three blue arrows) and (Co, Ni) atoms along the (200) plane if viewing with the [110] zone axis. Three arrows indicate the (200) planes occupied by red V atoms in the fcc-$\mathrm{L1_2}$ plate. Two green lines indicate the hcp lattice of 2 atom-layer thick. Importantly, this hcp-structured $\mathrm{L1_2}$ is sandwiched by two fcc-$\mathrm{L1_2}$ layers (one is the fcc-twin), indicating the presence of a small number of hcp structure inside the fcc plate. (**c, d**) Corresponding Co-map and Ni-map.

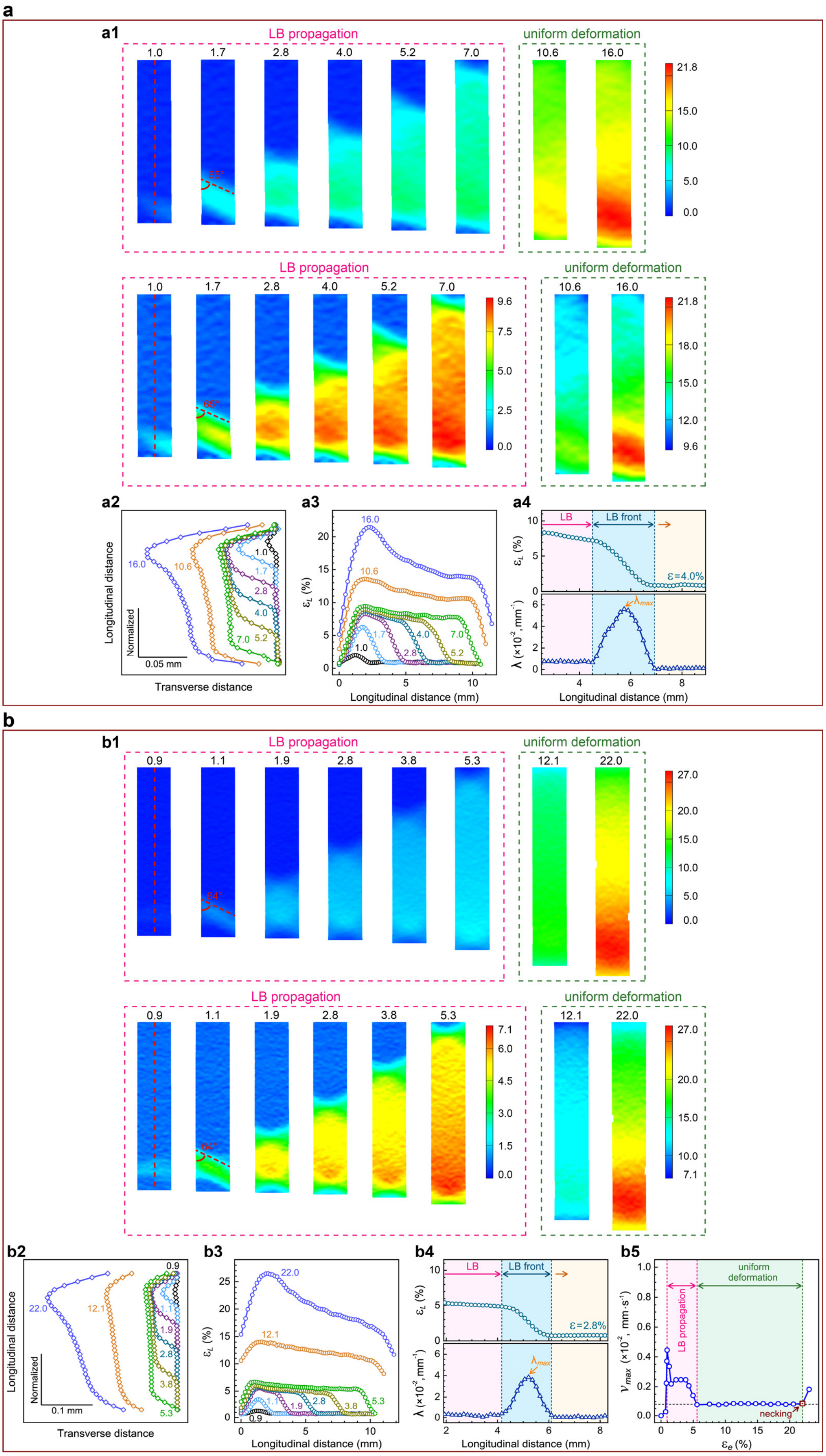

a
a1
LB propagation
uniform deformation
1.0
1.7
2.8
4.0
5.2
7.0
10.6
16.0
21.8
15.0
10.0
5.0
0.0
65°
9.6
7.5
5.0
2.5
0.0
18.0
12.0
a2
Longitudinal distance
Normalized
0.05 mm
Transverse distance
a3
ε_L (%)
Longitudinal distance (mm)
a4
LB
LB front
ε=4.0%
λ (×10^{-2}, mm^{-1})
λ_{max}
Longitudinal distance (mm)
b
b1
LB propagation
uniform deformation
0.9
1.1
1.9
2.8
3.8
5.3
12.1
22.0
27.0
20.0
15.0
10.0
5.0
0.0
64°
7.1
6.0
4.5
3.0
1.5
b2
Longitudinal distance
Normalized
0.1 mm
Transverse distance
b3
ε_L (%)
Longitudinal distance (mm)
b4
LB
LB front
ε=2.8%
λ (×10^{-2}, mm^{-1})
λ_{max}
Longitudinal distance (mm)
b5
LB propagation
uniform deformation
necking
v_{max} (×10^{-2}, mm·s^{-1})
ε_e (%)

Supplementary Fig. 2 | Two-dimensional full-field strain measurements during tensile deformation in two micro-structures of differing yield strengths by the digital-image-correlation (DIC) in situ tensile testing. (**a**) Results in the microstructure M_4 (**Fig. 1a**) of yield strength of 2 GPa (**Fig. 2a**). **a1**: Strain contours in the gauge section along the longitudinal direction, showing the strain and its evolution in the tensile direction (ε_L). Number above each map: tensile strain ε_{app} (%). Scale bar (color): range of ε_L, with maximal and minimal ε_L at both ends. The first two images show that LB begins to nucleate at strain of 1.0% soon after yield-drop, with an angle of 65 degree relative to tensile axis. Upper/lower: Strain profiles, respectively, with one/two fixed strain legends. **a2**: Formation and evolution of local lateral shrinkage with increasing ε_{app} (number %). **a3**: Heterogeneous distribution of ε_L along dash line in the leftmost image in (**a1**). **a4**: Distribution of strain (upper panel) and strain gradient (lower panel) in the LB front region at ε_{app} of 4%. (**b**) Corresponding results (**b1-b4**) in the microstructure M_7 of yield strength of 1.6 GPa (**Extended data Fig. 1a**). **b5**: Change of v_{max} with tensile strain. Note the initial drop in v_{max} as compared to that as shown in Fig. 3a in M_4 with yield strength of 2 GPa.

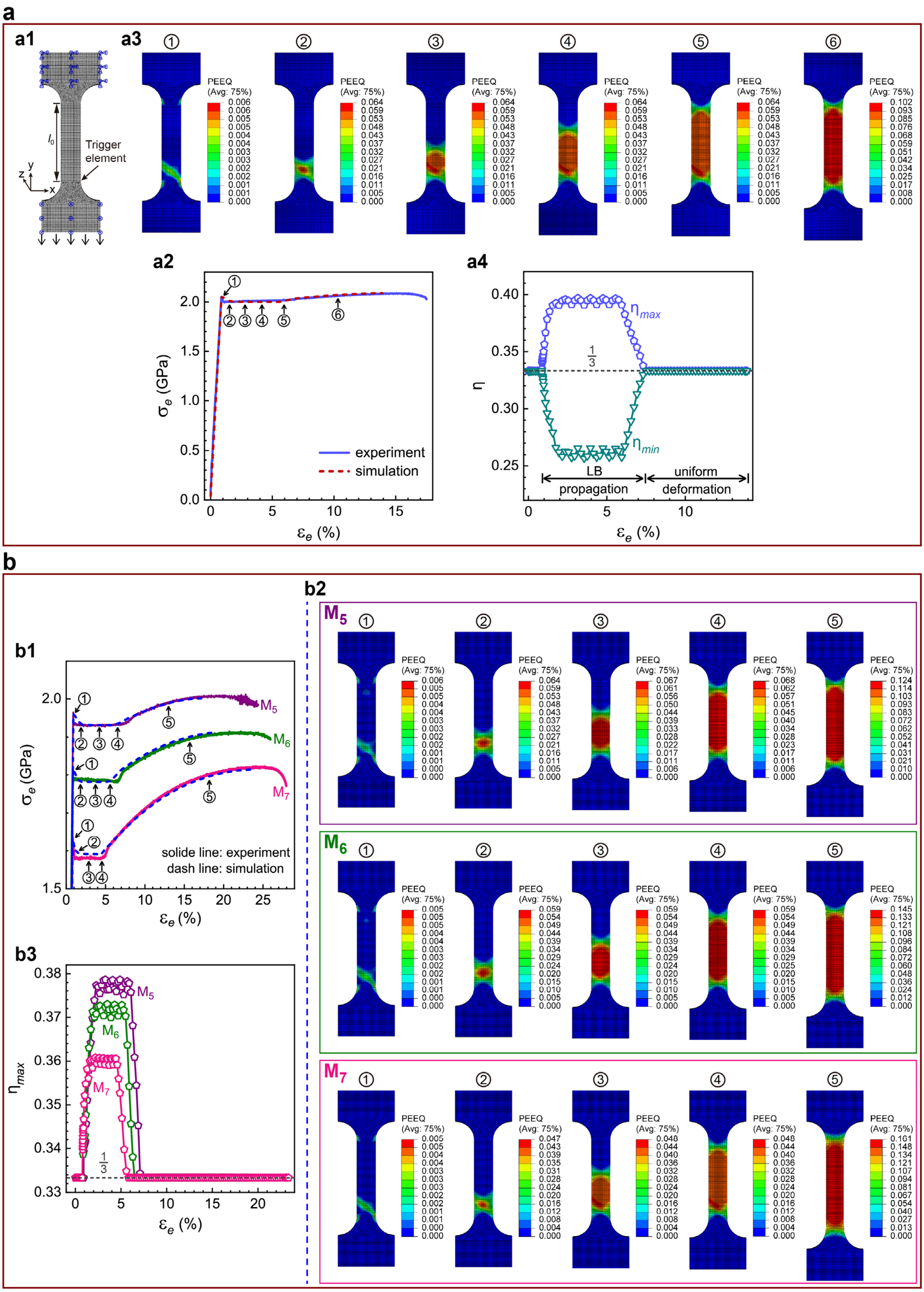
a
a1
l_0
Trigger element
a3
PEEQ (Avg: 75%)
a2
experiment
simulation
σ_e (GPa)
ε_e (%)
a4
η_{max}
η_{min}
1/3
LB propagation
uniform deformation
η
ε_e (%)
b
b1
M_5
M_6
M_7
solide line: experiment
dash line: simulation
σ_e (GPa)
ε_e (%)
b2
M_5
M_6
M_7
PEEQ (Avg: 75%)
b3
η_{max}
ε_e (%)

Supplementary Fig. 3 | FEM calculations and simulations of tensile deformation in various microstructures. (**a**) Results for the microstructure (M_4, **Fig. 1a**) with yield strength of 2 GPa as shown in **Fig. 2a**. **a1**: Specimen shape, elements, boundary, and loading conditions for FEM simulations. **a2**: Simulated stress-strain curve (red dash line), which well reproduces the characteristic tensile responses in the measured curve (blue line), including yield-drop, LB, and uniform deformation. **a3**: Equivalent plastic strain (PEEQ) contours corresponding to five points in the simulated curve in **a2**. Scale bar (color): range of PEEQ, with maximal and minimal values at both ends. **a4**: Maximal (η_{max}) and minimal stress triaxiality parameter (η_{min}) that keep constant 0.39 and 0.26 at the LB front during the LB propagation. Dash line: constant η of 1/3 during uniform deformation. (**b**) Corresponding results for the microstructures from M_5 to M_7 as shown in **Extended data Fig. 1a. b1**: Three simulated stress-strain curves (dash lines). The corresponding measured curves (solid lines) are shown for comparison. **b2**: Three groups of PEEQ contours respectively. **b3**: η_{max} as a function of tensile strain. Note the decrease in η_{max} with decreasing yield strength.

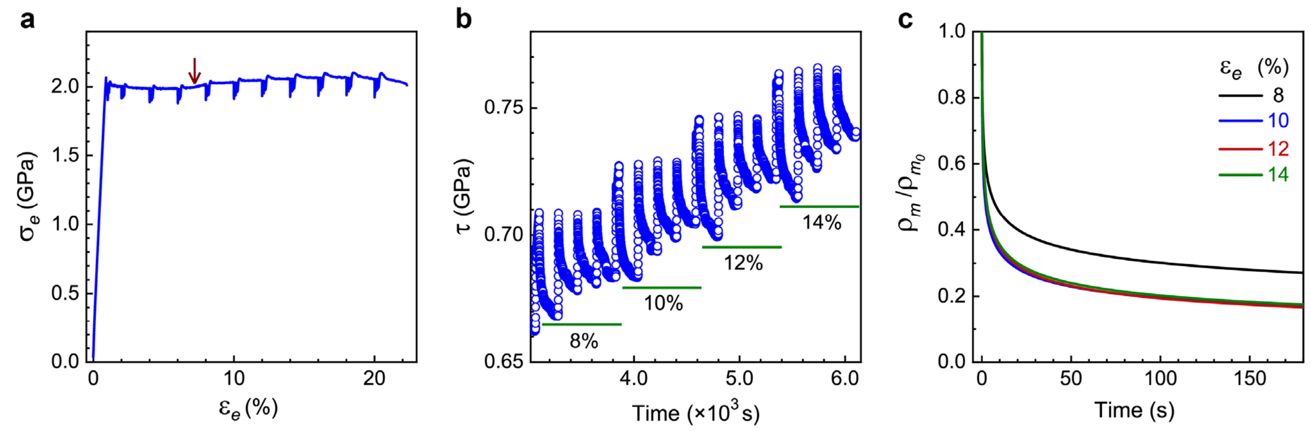

Supplementary Fig. 4 | Measurement of mobile dislocation density in the microstructure (Fig. 1a) of yield strength of 2 GPa (Fig. 2a) by the stress relaxation tensile testing. (**a**) Tensile engineering stress-strain ($\sigma_e - \varepsilon_e$) curve. Arrow: the end of Lüders band extension. (**b**) Shear stress (τ) *vs* relaxation time curve. Number: starting strain for relaxation. (**c**) Evolution of the mobile dislocation density ($^{\rho_m}/_{\rho_{m_0}}$) with relaxation time (totally 180 seconds) during the first relaxation cycle.

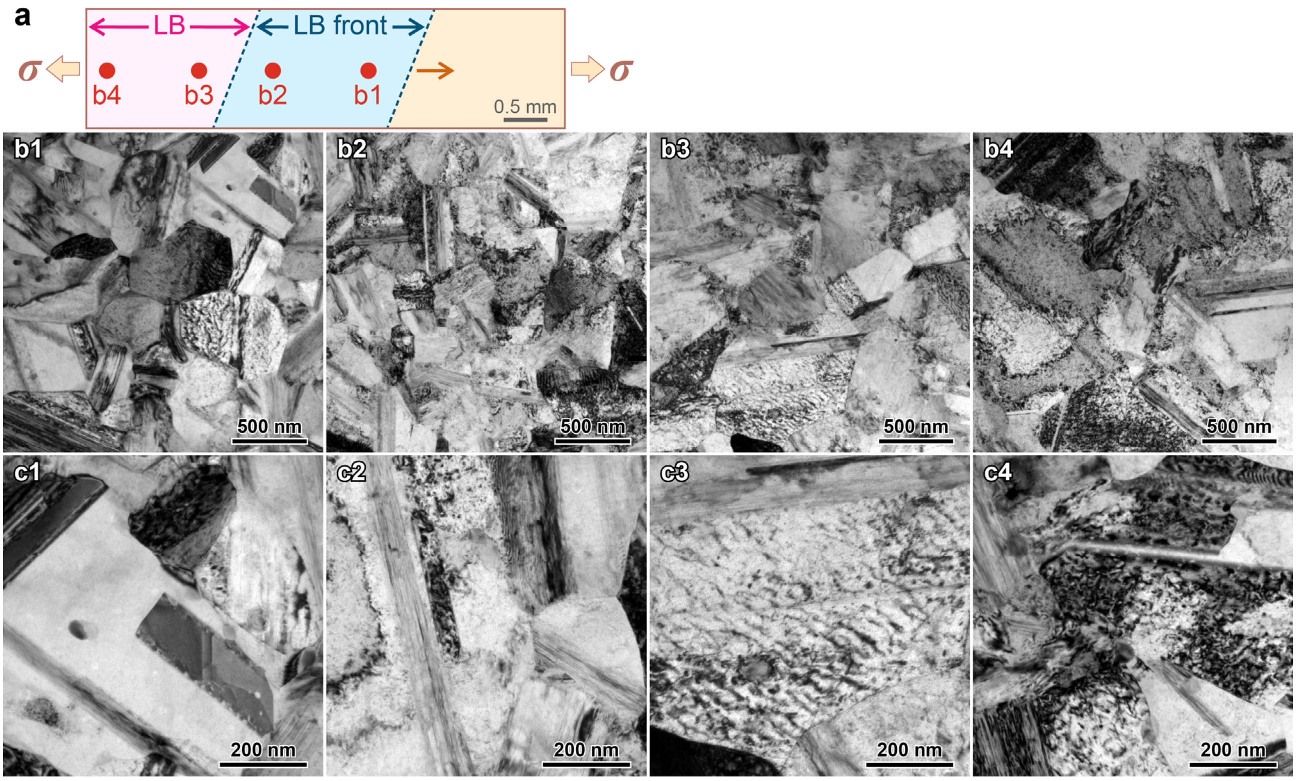

Supplementary Fig. 5 | Dislocation production at the site-specific locations during the LB propagation by TEM observations. (**a**) Schematic four sites in the gauge section where thin TEM foils were cut off from the sample during an interrupted tensile deformation at strain of 5% by means of the focused ion beam (FIB) method. Arrow: the LB propagating direction. σ: applied stress. (**b**) Dislocation production at various locations. **b1**: Low dislocation density in almost half grains. **b2-b4**: Increased dislocation density away from the LB front. (**c**) Locally enlarged images corresponding to **b1-b4** respectively.

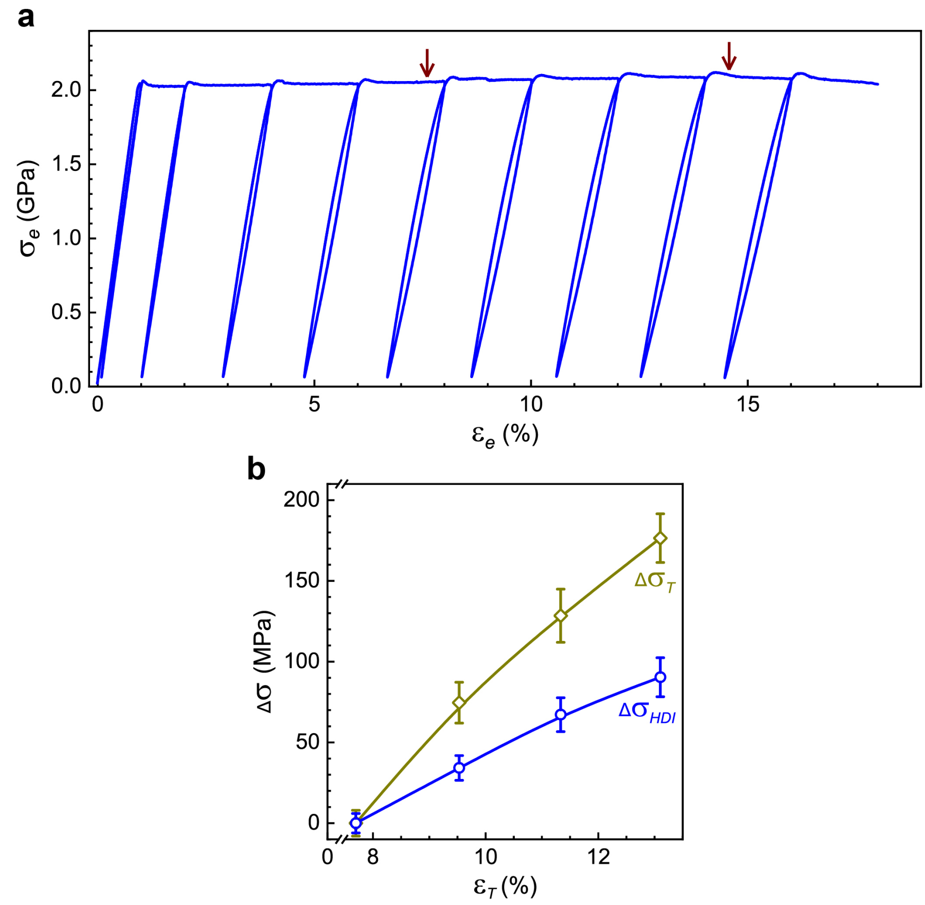

Supplementary Fig. 6 | Measurement of HDI stress (σ_{HDI}) by the unload-reload tensile testing at room temperature in the $\mathbf{M_4}$ microstructure of yield strength of 2 GPa (Fig. 2a). (**a**) Engineering stress-strain ($\sigma_e - \varepsilon_e$) curve, showing the mechanical hysteresis loop at each unload-reload cycle. Arrows: the end of Lüders extension (the first) and of uniform deformation (the second). (**b**) Increment of HDI-stress ($\Delta\sigma_{HDI}$) and of flow stress ($\Delta\sigma_T$) as a function of true strain during uniform deformation. The mean values ± standard error of the mean are presented based on three unload-reload tensile tests.

References

1. Ding, Q., Zhang, Y., Chen, X., Fu, X., Chen, D., Chen, S., Gu, L., Wei, F., Bei, H., Gao, Y., Wen, M., Li, J., Zhang, Z., Zhu, T., Ritchie, R. O. & Yu, Q. Tuning element distribution, structure and properties by composition in high-entropy alloys. *Nature* **574**, 223-227 (2019).
2. Chen, X. F., Wang, Q., Cheng, Z. Y., Zhu, M. L., Zhou, H., Jiang, P., Zhou, L. L., Xue, Q. Q., Yuan, F. P., Zhu, J., Wu, X. L. & Ma, E. Direct observation of chemical short-range order in a medium-entropy alloy. *Nature* **592**, 712-716 (2021).
3. Johnston, W. G. & Gilman, J. J. Dislocation velocities, dislocation densities, and plastic flow in lithium fluoride crystals. *J. Appl. Phys.* **30**, 129-144 (1959).
4. Antolovich, S. D. & Armstrong, R. W. Plastic strain localization in metals: origins and consequences. *Prog. Mater Sci.* **59**, 1-160 (2014).
5. Gao, S., Bai, Y., Zheng, R. X., Tian, Y. Z., Mao, W., Shibata, A. & Tsuji, N. Mechanism of huge Lüders-type deformation in ultrafine grained austenitic stainless steel. *Scr. Mater.* **159**, 28-32 (2019).
6. Yuan, F. P., Yan, D. S., Sun, J. D., Zhou, L. L., Zhu, Y. T. & Wu, X. L. Ductility by shear band delocalization in the nano-layer of gradient structure. *Mater. Res. Lett.* **7**, 12-17 (2019).
